# Supplementary material for: Retinal nerve fibre layer thickness reflects characteristics of brain grey and white matter
Source: Imaging Neurosci (Camb). 2026 Apr 2;4:IMAG.a.1174. doi: 10.1162/IMAG.a.1174 (PMC13058853; doi:10.1162/IMAG.a.1174)
Supplement: Supplementary Figures [file IMAG.a.1174_supp1.pdf]

SUPPLEMENTARY FIGURES

VBM

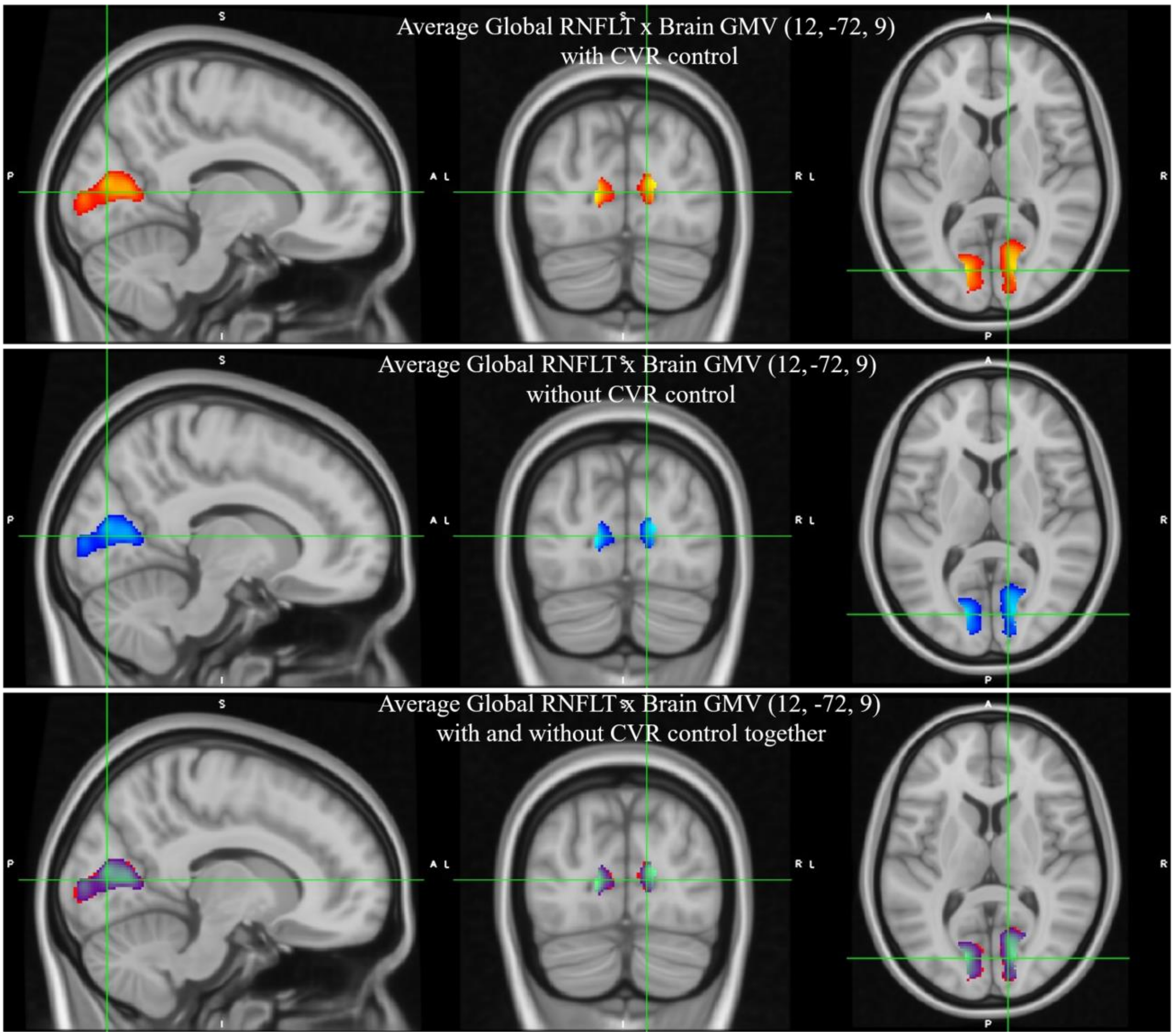

Figure S1. Average Global Mean RNFLT positive correlations with the brain GMD with and without CVR factors controlling (n=769). **Upper** figure shows the correlations when controlling for the CVR factors i.e., BMI, LDL and HDL Cholesterol scores and Diabetes, Hypertension, Smoking and Physical Activity status (red) in addition to age, sex, total intracranial volume and retina scan radius. **Middle** figure shows the correlations without CVR factor controlling, only age, sex, total intracranial volume and retina scan radius were controlled (blue)**.** Lower figure shows both correlations together (overlap, purple). Results shown on MNI152_T1_05.mm template, corrected at cluster-level pFWE<0.05 and uncorrected at voxel-level p<0.001. RNFLT: retinal nerve fibre layer thickness, GMD: grey matter density.

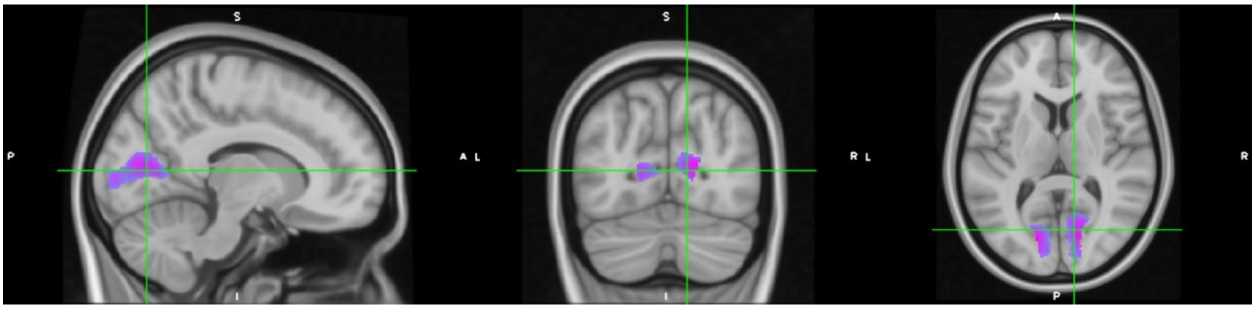

Figure S2. Conjunction (overlap) between Average Global Mean RNFLT positive correlations with and without CVR when age, sex, TIV and average retina scan radius were controlled

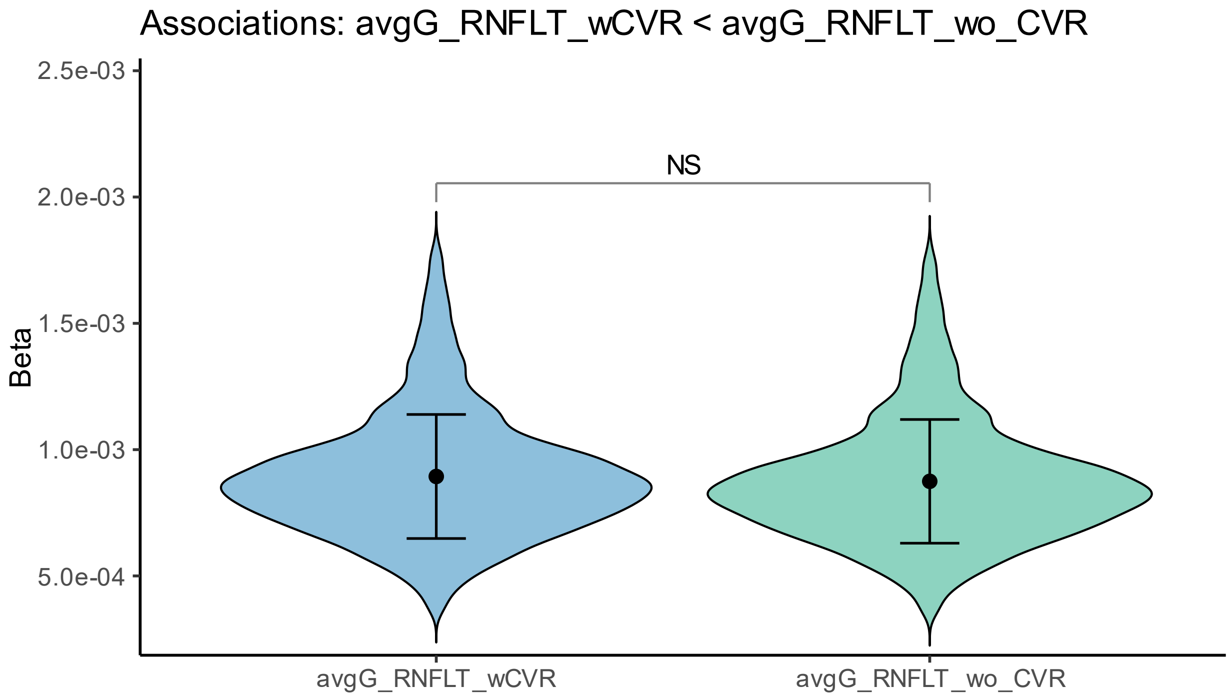

Figure S3. Comparison of the Beta values of the overlapped regions between Average Global Mean RNFLT positive correlations **with** and **without** controlling for the CVR factors in addition to age, sex, TIV and average retina scan radius variables. The one-sided paired-samples t-test results were not statistically significant between the beta values [T (2777) = 98.1, estimate= 0.0000192, p=1, cohen's_d = 1.81].

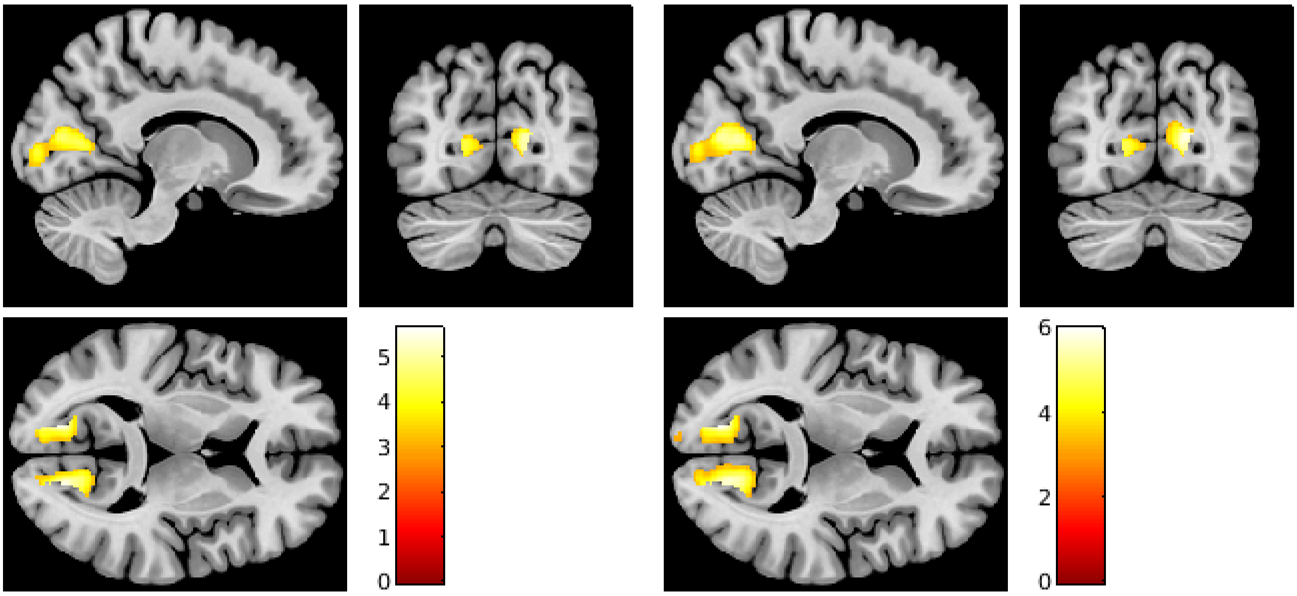

Figure S4. **Left** (left side) **and Right** (right side) **Global Mean RNFLT** positive correlations with the brain GMV when controlling for age, sex, total intracranial volume, retina scan radius and CVR factors i.e., BMI, LDL and HDL Cholesterol scores and Diabetes, Hypertension, Smoking and Physical Activity status **(n=769).** Results shown on MNI registered CAT-T1_IXI 555 GS standard atlas, Neurological View. Coordinates (12, -72, 9). Color bar shows T statistics corrected at cluster-level pFWE<0.05 and uncorrected at voxel-level p<0.001. RNFLT: Retinal Nerve Fiber Layer Thickness, GMV: Gray Matter Volume.

Comparison of the Left Global RNFLT Correlations to the Right Global RNFLT Correlations when CVR factors were and were not taken into account in addition to age, sex, TIV and Retina Scan Radius variables:

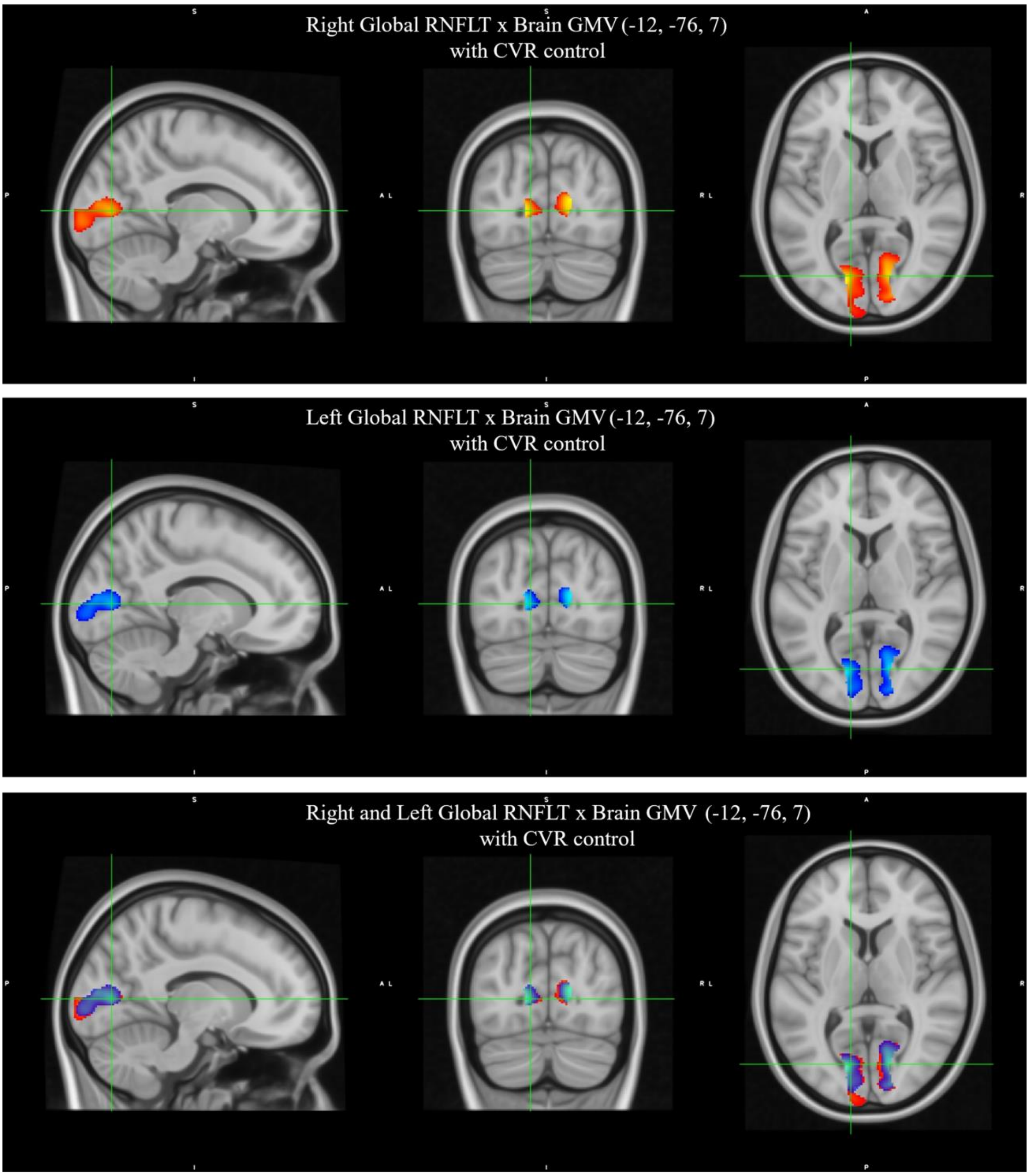

Figure S5. Comparison of the **Right** and **Left Global Mean RNFLT** positive correlations with the brain GMV when controlling for age, sex, total intracranial volume, retina scan radius and **CVR** factors i.e., BMI, LDL and HDL Cholesterol scores and Diabetes, Hypertension, Smoking and Physical Activity status **(n=769).** Results shown, at cluster-level corrected $p < 0.05$ for FWER with an uncorrected $p < 0.001$ voxel-level clustering threshold, on MNI152_T1_0.5mm standard atlas, Neurological View. RNFLT: Retinal Nerve Fiber Layer Thickness

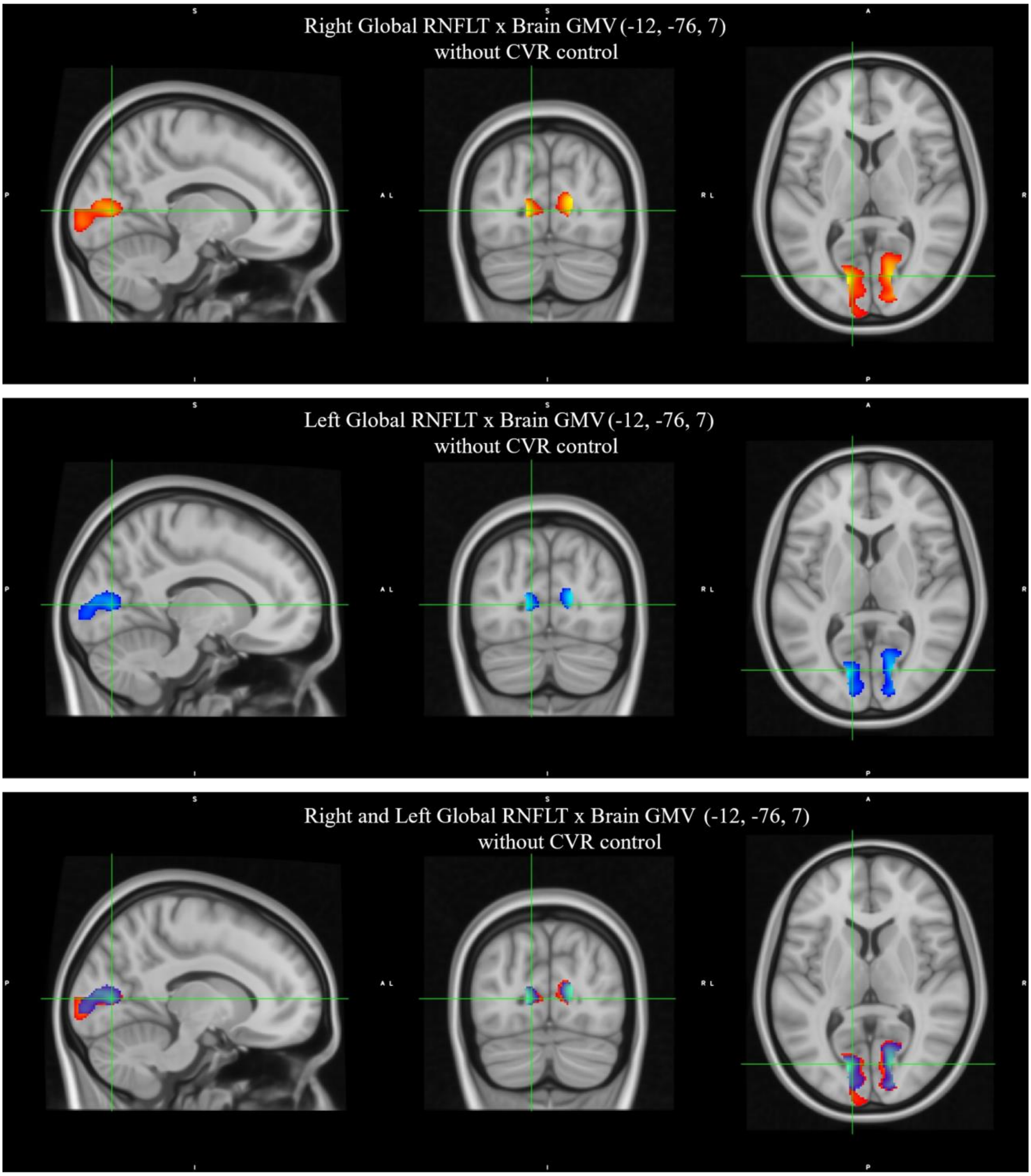

Figure S6. Comparison of the **Right** and **Left Global Mean RNFLT** positive correlations with the brain GMV when controlling for only age, sex, total intracranial volume and retina scan radius **(n=769).** Results shown, at cluster-level corrected $p < 0.05$ for FWER with an uncorrected $p < 0.001$ voxel-level clustering threshold, on MNI152_T1_0.5mm standard atlas, Neurological View. RNFLT: Retinal Nerve Fiber Layer Thickness

Comparison of the Right Global RNFLT Correlations when CVR factors were and were not taken into account in addition to age, sex, TIV and Retina Scan Radius variables:

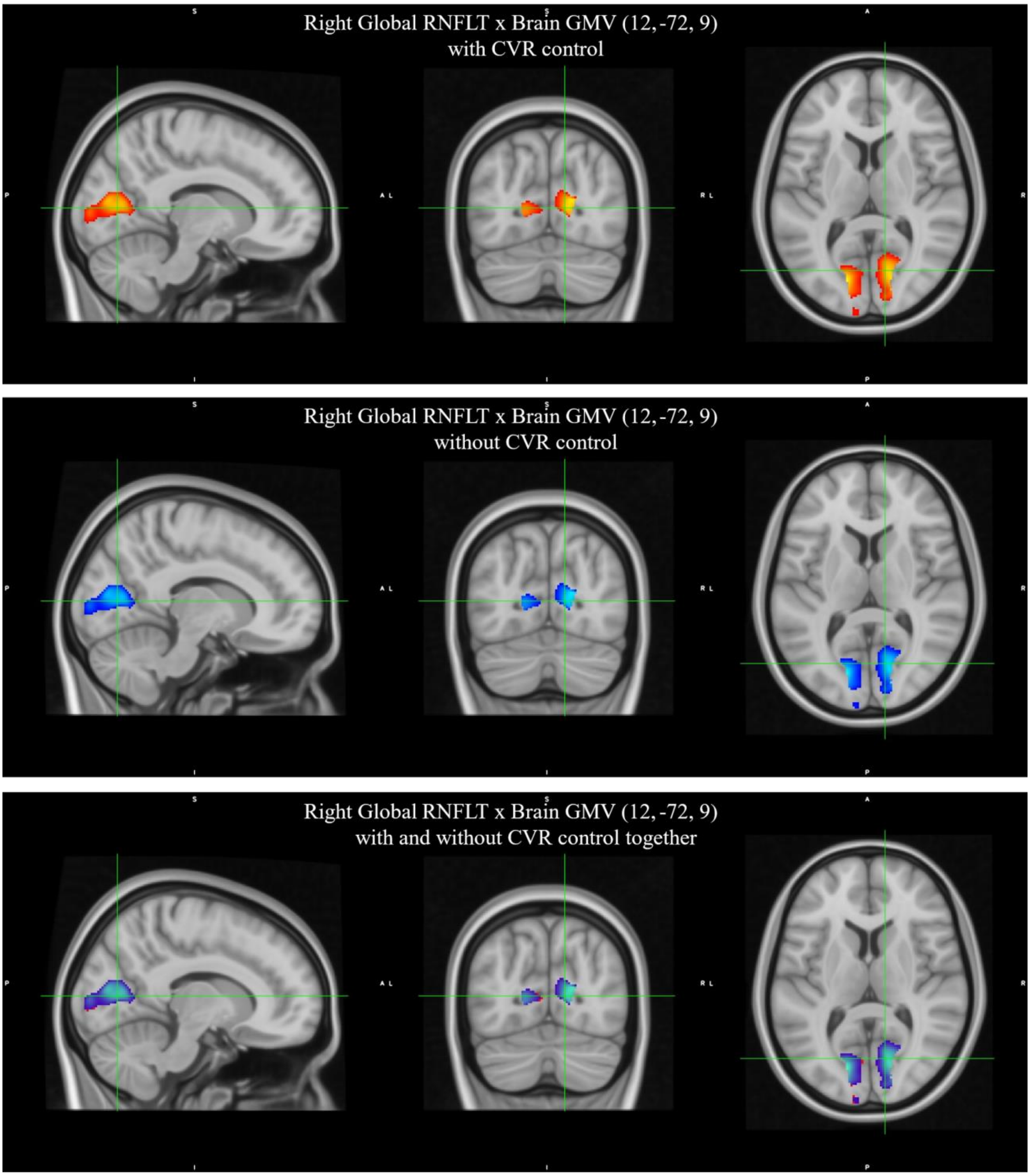

Figure S7. **Right** Global Mean RNFLT positive correlations with the brain GMV **with and without CVR factors** controlling (n=769). **Upper** figure shows the correlations when controlling for the CVR factors i.e., BMI, LDL and HDL Cholesterol scores and Diabetes, Hypertension, Smoking and Physical Activity status (red) in addition to age, sex, total intracranial volume and retina scan radius. **Middle** figure shows the correlations when controlling for only age, sex, total intracranial volume and related retina scan radius (blue)**.** Lower figure shows both correlations together (overlap, purple). Results shown on MNI152_T1_0.5mm template, corrected at cluster-level pFWE<0.05 and uncorrected at voxel-level p<0.001. RNFLT: Retinal Nerve Fiber Layer Thickness, GMV: Gray Matter Volume

Comparison of the Right Global RNFLT Correlations when CVR factors were and were not taken into account in addition to age, sex, TIV and Retina Scan Radius variables:

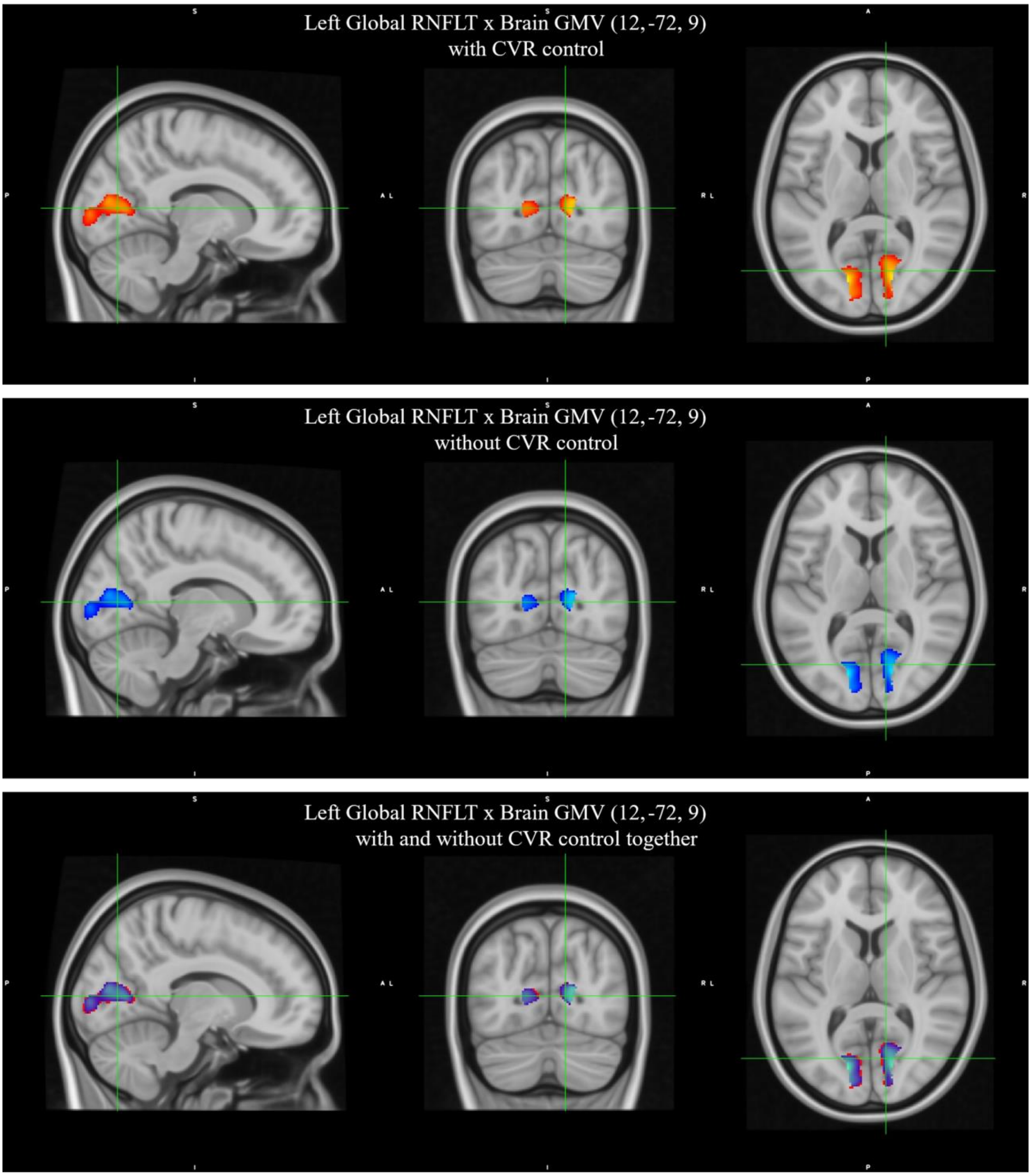

Figure S8. **Left** Global Mean RNFLT positive correlations with the brain GMV **with and without CVR factors** controlling (n=769). **Upper** figure shows the correlations when controlling for the CVR factors i.e., BMI, LDL and HDL Cholesterol scores and Diabetes, Hypertension, Smoking and Physical Activity status (red) in addition to age, sex, total intracranial volume and retina scan radius. **Middle** figure shows the correlations when controlling for only age, sex, total intracranial volume and related retina scan radius (blue)**.** Lower figure shows both correlations together (overlap, purple). Results shown on MNI152_T1_0.5mm template, corrected at cluster-level pFWE<0.05 and uncorrected at voxel-level p<0.001. RNFLT: Retinal Nerve Fiber Layer Thickness, GMV: Gray Matter Volume

TBSS: FRACTIONAL ANISOTROPY

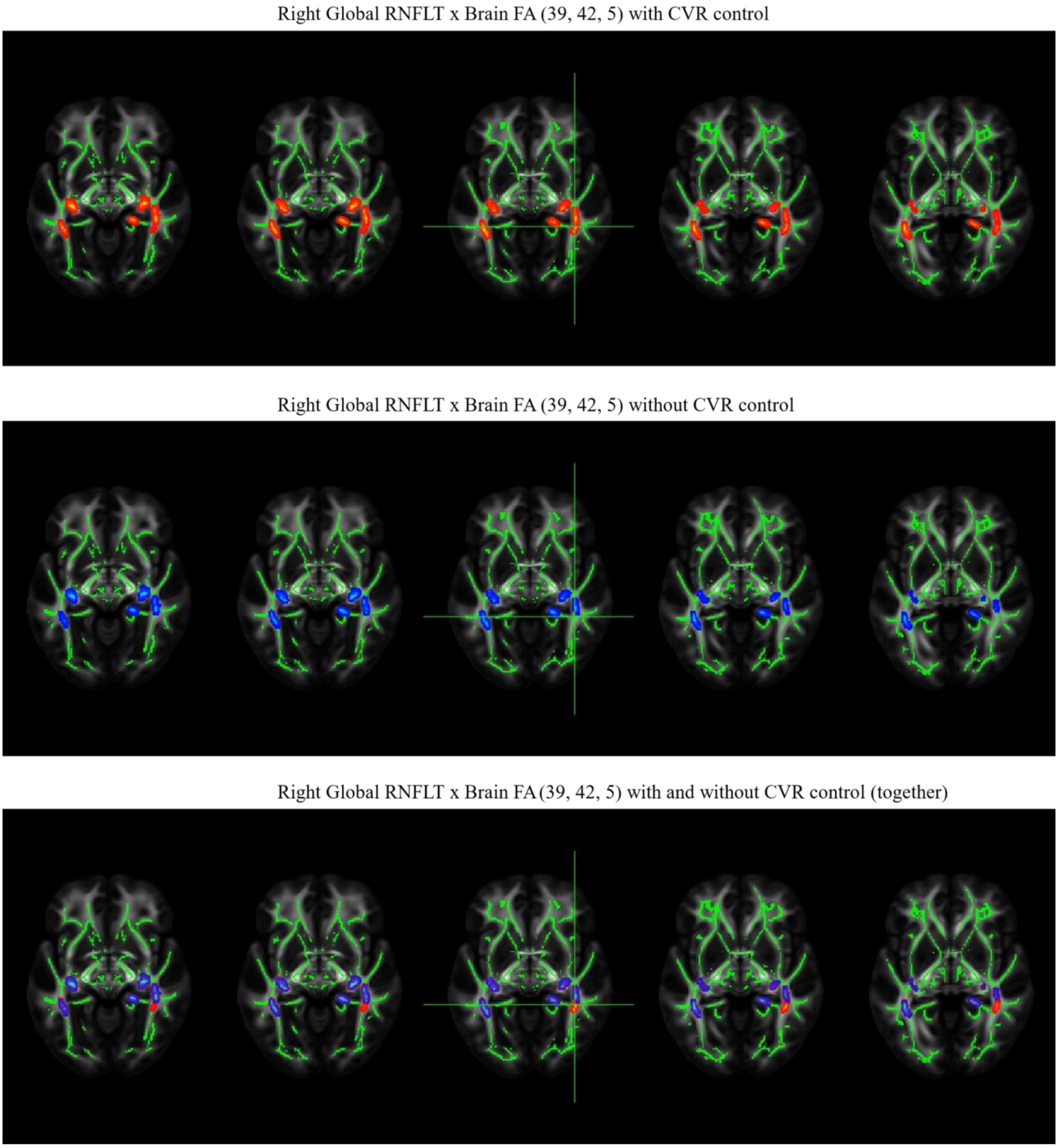

Figure S9. **Right** Global Mean RNFLT positive correlations with brain Fractional Anisotropy when controlling for the CVR factors i.e., BMI, LDL and HDL Cholesterol scores and Diabetes, Hypertension, Smoking and Physical Activity status (**i.e., with CVR**) in addition to age, sex and retina scan radius (**i.e., without CVR**), n=550. Results shown, at cluster-level corrected p< 0.05 for FWER with an uncorrected p<0.001 voxel-level clustering threshold, on MNI-registered FSL_HCP1065_FA_1mm standard atlas, Neurological View. RNFLT: Retinal Nerve Fiber Layer Thickness

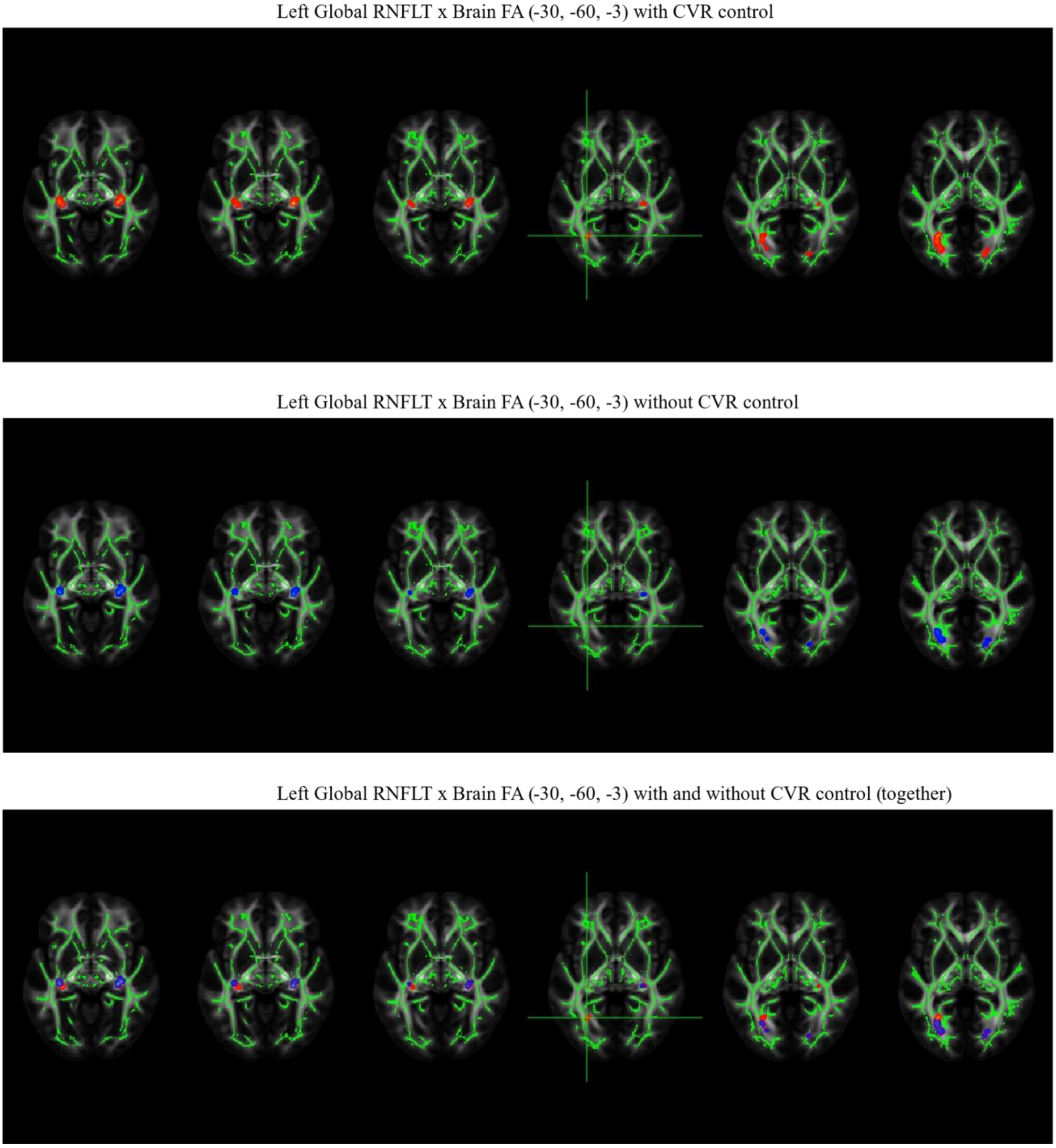

Figure S10. **Left** Global Mean RNFLT positive correlations with brain Fractional Anisotropy when controlling for the CVR factors i.e., BMI, LDL and HDL Cholesterol scores and Diabetes, Hypertension, Smoking and Physical Activity status (**i.e., with CVR**) in addition to age, sex and retina scan radius (**i.e., without CVR**), n=550. Results shown, at cluster-level corrected $p < 0.05$ for FWER with an uncorrected $p < 0.001$ voxel-level clustering threshold, on MNI-registered FSL_HCP1065_FA_1mm standard atlas, Neurological View. RNFLT: Retinal Nerve Fiber Layer Thickness

Left Global RNFLT x Brain FA (-29, -58, -1)

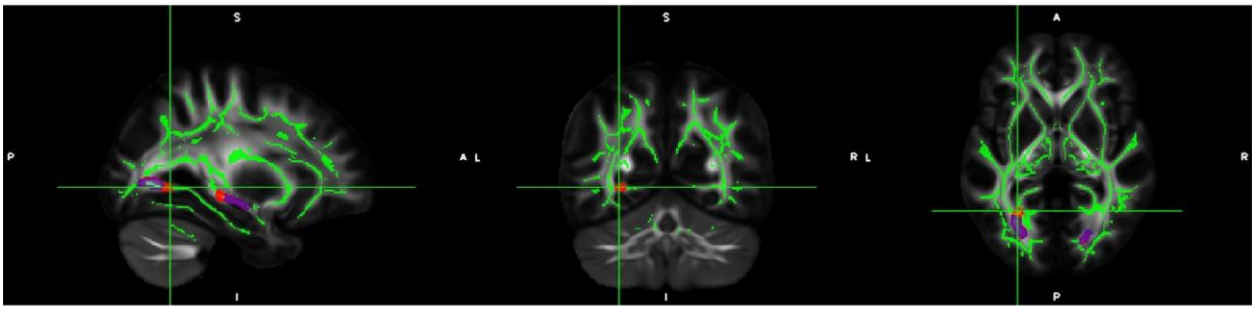

Right Global RNFLT x Brain FA (40, -42, -5)

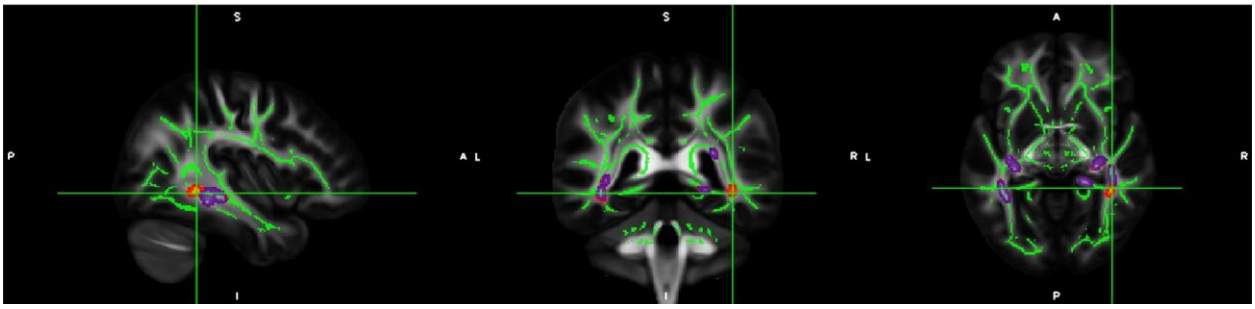

Figure S11. Left Global (upper side) and Right Global (lower side) Mean RNFLT positive correlations with brain Fractional Anisotropy. **Blue** shows the results when controlling for only age, sex, retina scan radius (**i.e., without CVR**) and **red** shows the results when controlling CVR factors additionally (**i.e., with CVR**), and **purple** shows the overlap between blue and red colors (n=550). Results shown, at cluster-level corrected $p < 0.05$ for FWER with an uncorrected $p < 0.001$ voxel-level clustering threshold, on MNI-registered FSL_HCP1065_FA_1mm standard atlas, Neurological View. RNFLT: Retinal Nerve Fiber Layer Thickness

Comparison of the Left and Right Global RNFLT positive correlations with the Brain Fractional Anisotropy, n=550, with and without CVR factors:

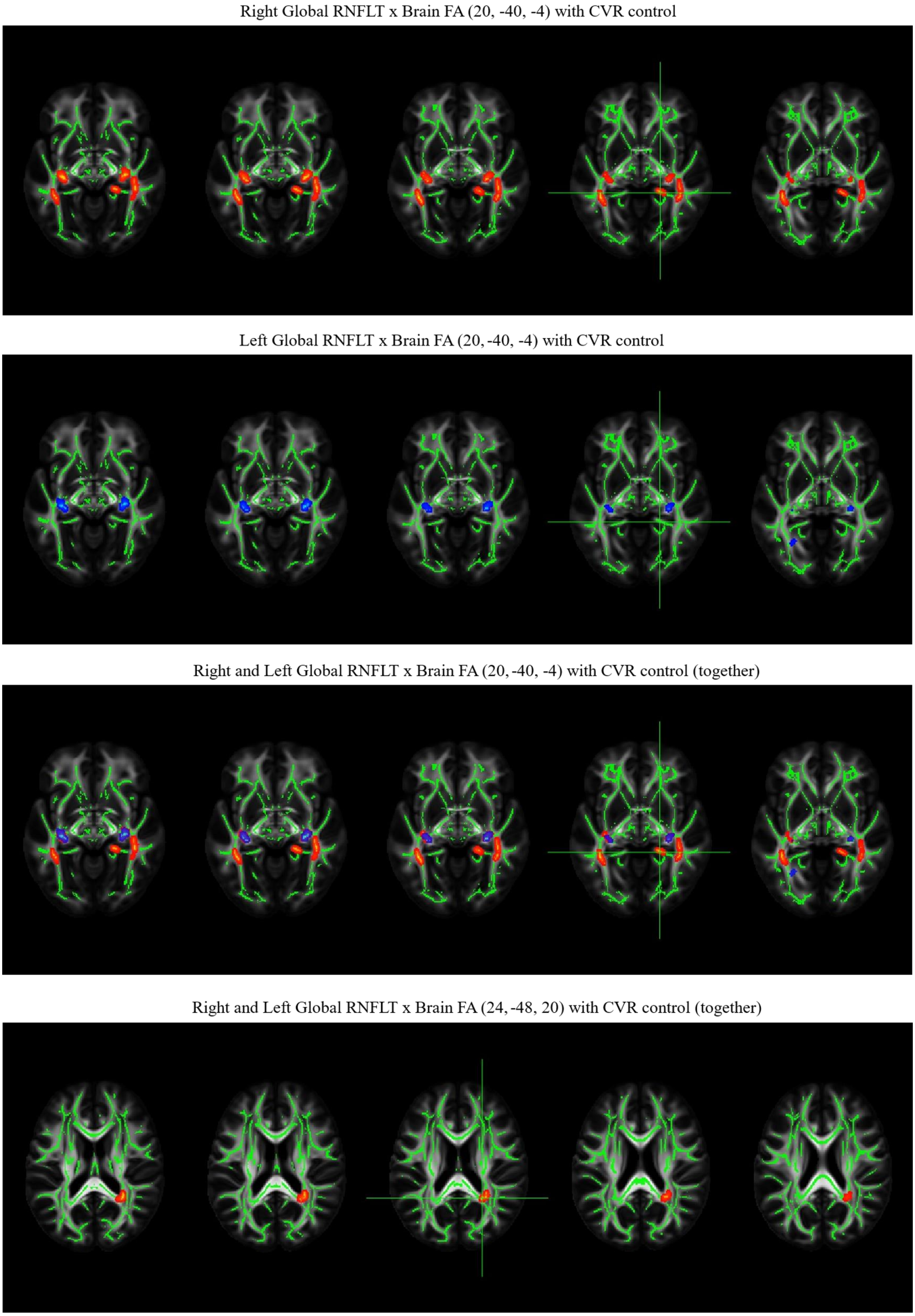

Figure S12. Comparison of the **Right** and **Left Global Mean RNFLT** positive correlations with the brain FA when controlling for the CVR factors i.e., BMI, LDL and HDL Cholesterol scores and Diabetes, Hypertension, Smoking

and Physical Activity status (**i.e., with CVR**) in addition to age, sex and regarding retina scan radius **(n=550).** Results shown, at cluster-level corrected p< 0.05 for FWER with an uncorrected p<0.001 voxel-level clustering threshold, on MNI-registered FSL_HCP1065_FA_1mm standard atlas, Neurological View. RNFLT: Retinal Nerve Fiber Layer Thickness, FA: Fractional Anisotropy

Right Global RNFLT x Brain FA (20, -40, -4) without CVR control

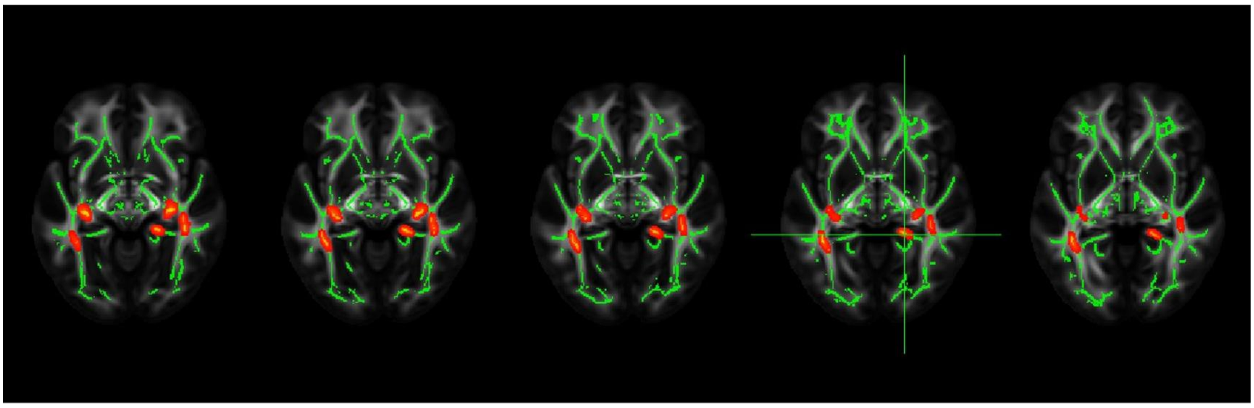

Left Global RNFLT x Brain FA (20, -40, -4) without CVR control

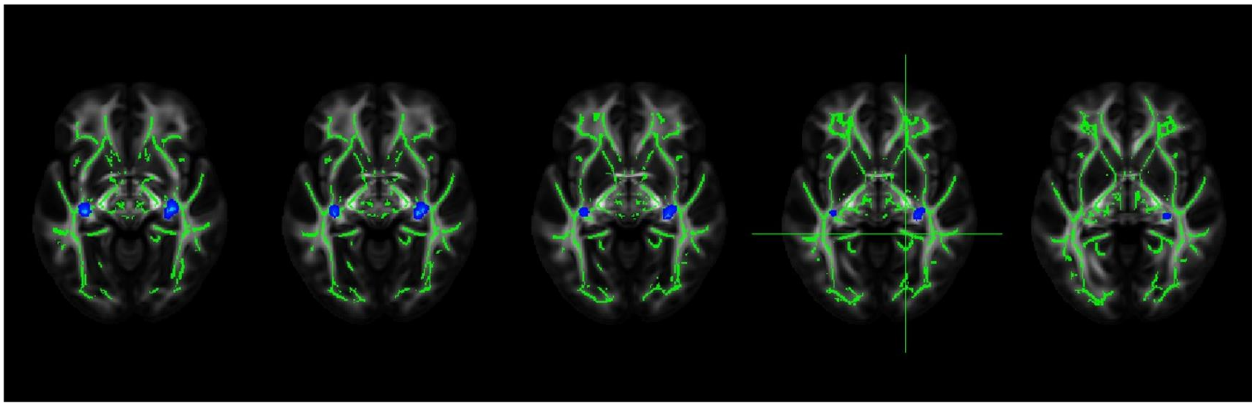

Right and Left Global RNFLT x Brain FA (20, -40, -4) without CVR control (together)

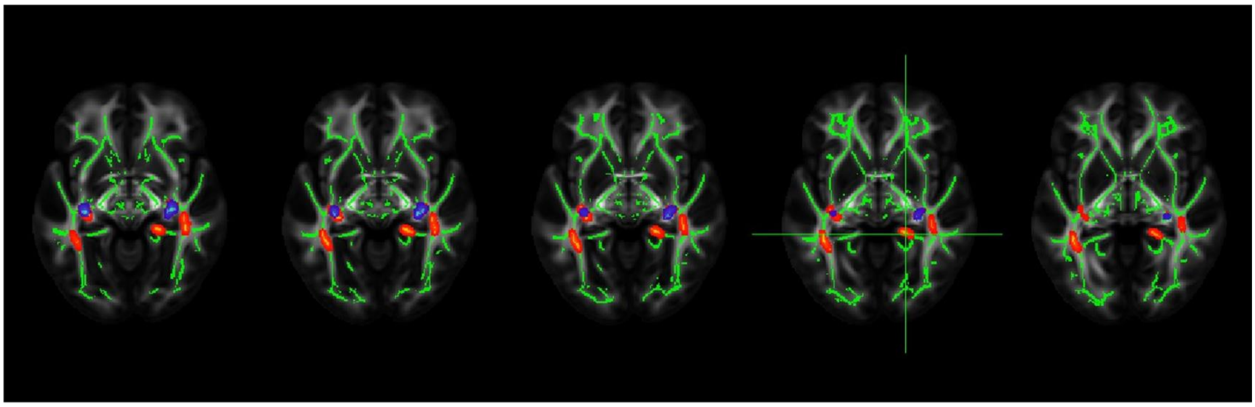

Right and Left Global RNFLT x Brain FA (24, -48, 20) without CVR control (together)

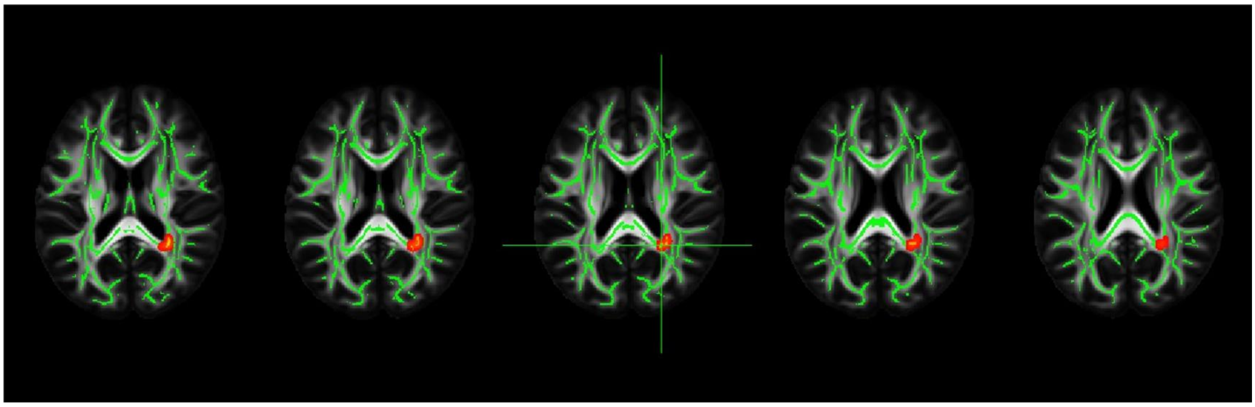

Figure S13. Comparison of the **Right** and **Left Global Mean RNFLT** positive correlations with the brain FA when controlling for only age, sex and regarding retina scan radius [(**i.e., without CVR, (n=550)].** Results shown, at cluster-level corrected p< 0.05 for FWER with an uncorrected p<0.001 voxel-level clustering threshold, on MNI-registered FSL_HCP1065_FA_1mm standard atlas, Neurological View. RNFLT: Retinal Nerve Fiber Layer Thickness, FA: Fractional Anisotropy

TBSS: MEAN DIFFUSIVITY

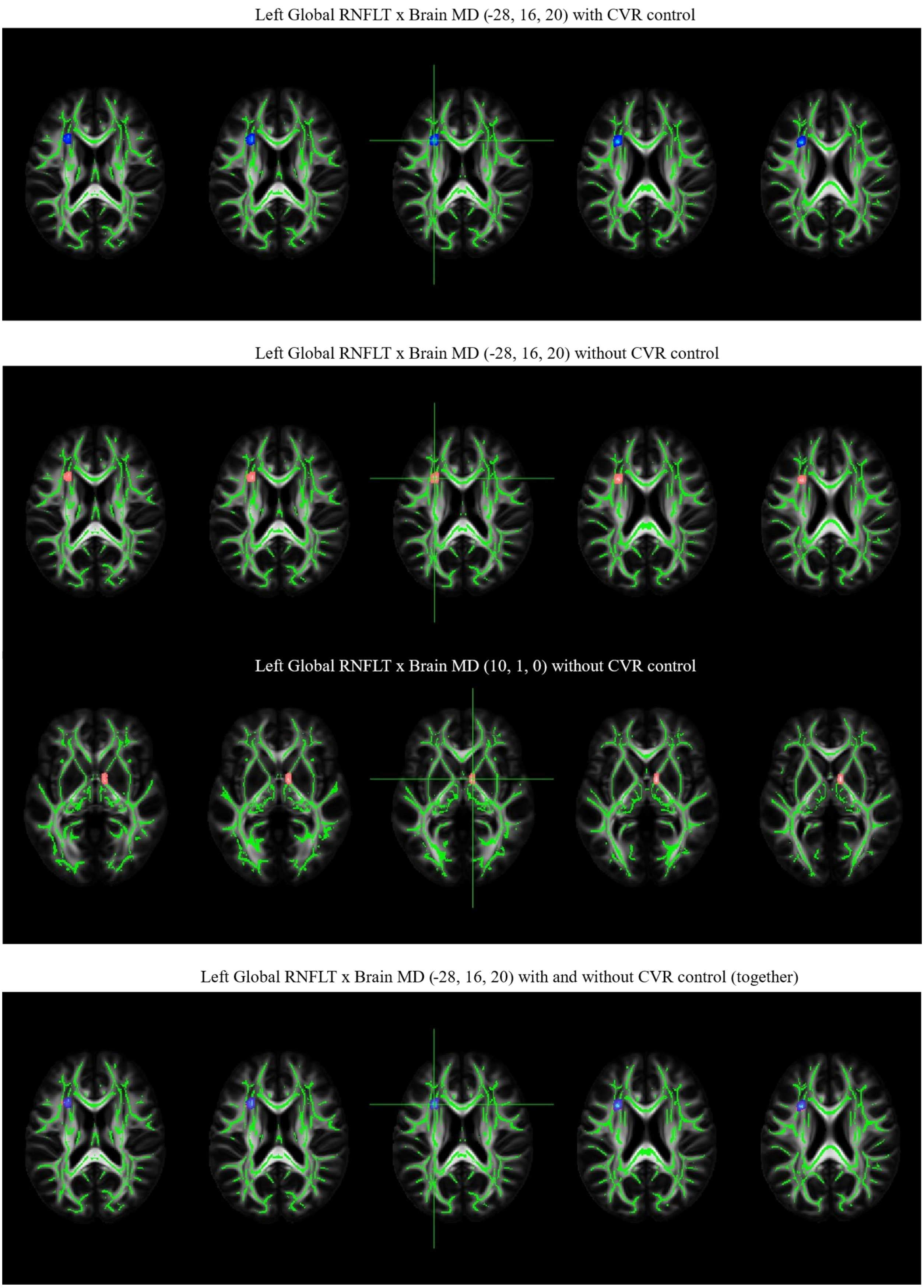

Figure S14. Left Global Mean RNFLT negative correlations with Mean Diffusivity **with and without CVR factors** controlling (n=550). **Upper** figure shows the correlations when controlling for the CVR factors i.e., BMI, LDL and HDL Cholesterol scores and Diabetes, Hypertension, Smoking and Physical Activity status (blue) in addition to age,

sex, total intracranial volume and retina scan radius. **Middle** figures show the correlations when controlling for only age, sex and related retina scan radius (pink)**. Lower** figure shows both correlations together (overlap, purple). Results shown, at cluster-level corrected p< 0.05 for FWER with an uncorrected p<0.001 voxel-level clustering threshold, on MNI-registered FSL_HCP1065_FA_1mm standard atlas, Neurological View. RNFLT: Retinal Nerve Fiber Layer Thickness

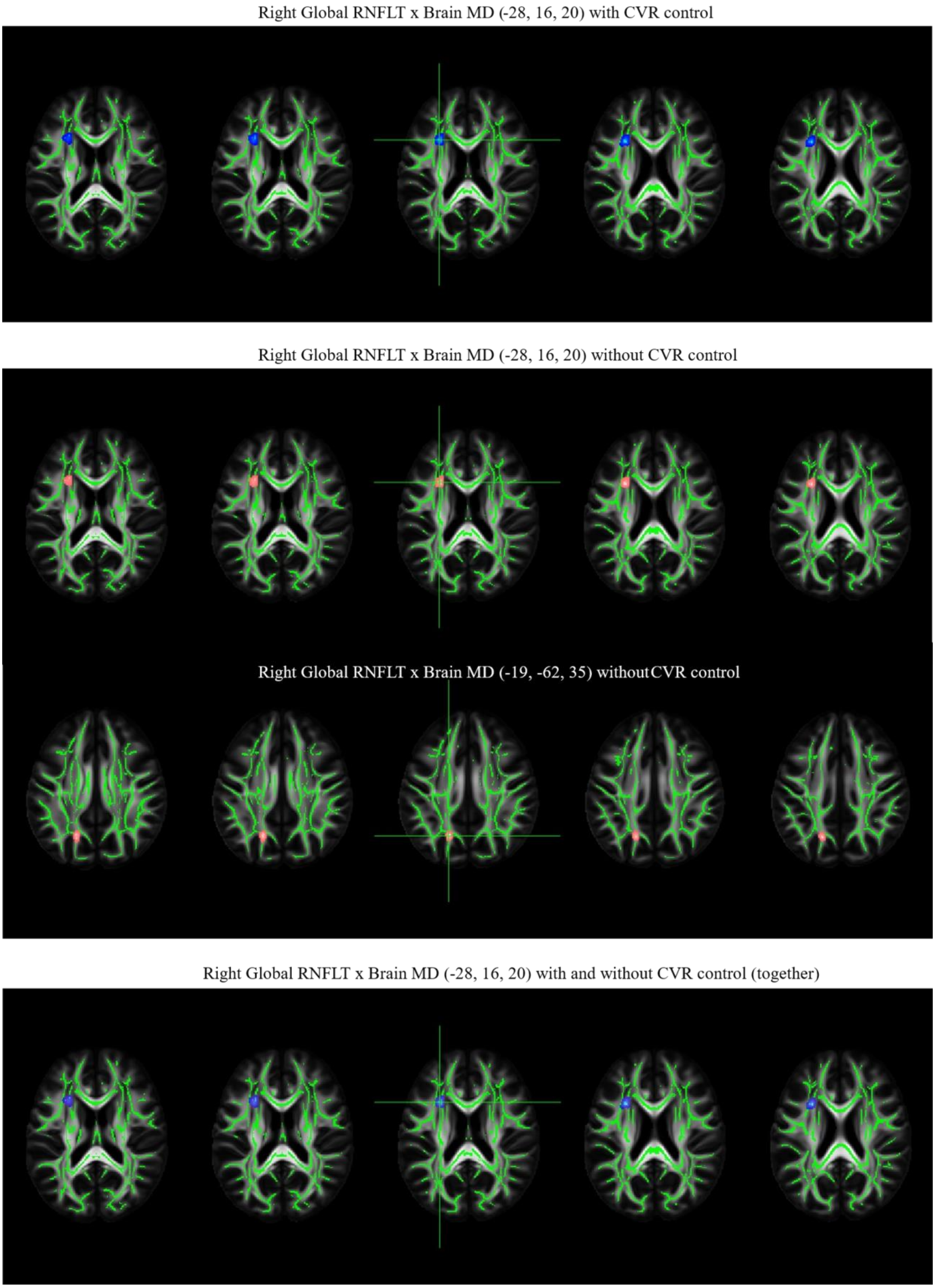

Figure S15. Right Global Mean RNFLT negative correlations with Mean Diffusivity **with and without CVR factors** controlling (n=550). **Upper** figure shows the correlations when controlling for the CVR factors i.e., BMI, LDL and HDL Cholesterol scores and Diabetes, Hypertension, Smoking and Physical Activity status (blue) in addition to age, sex, total intracranial volume and retina scan radius. **Middle** figures show the correlations when controlling for only

age, sex and related retina scan radius (pink)**. Lower** figure shows both correlations together (overlap, purple). Results shown, at cluster-level corrected p< 0.05 for FWER with an uncorrected p<0.001 voxel-level clustering threshold, on MNI-registered FSL_HCP1065_FA_1mm standard atlas, Neurological View. RNFLT: Retinal Nerve Fiber Layer Thickness

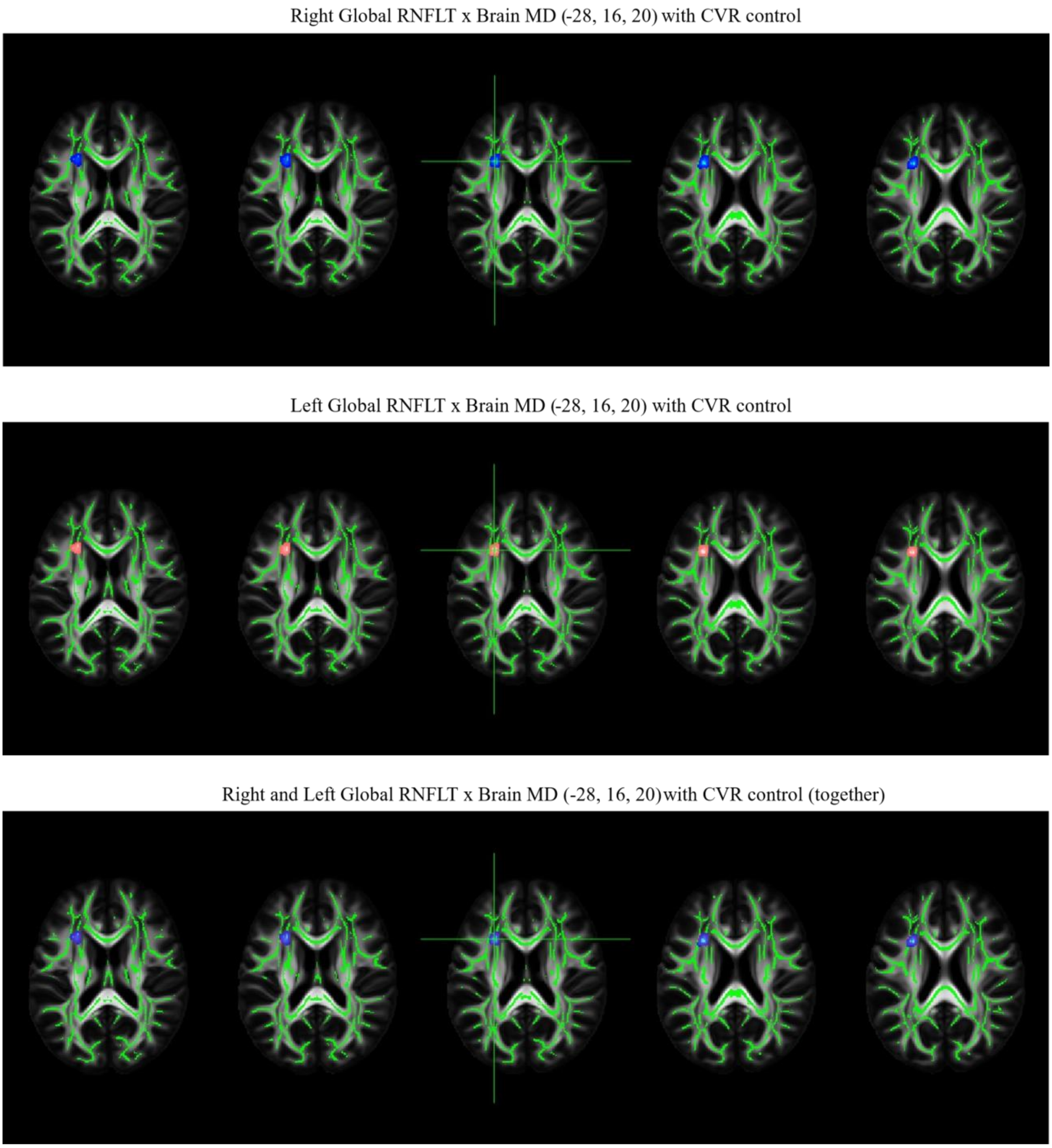

Figure S16. Comparison of the **Right** and **Left Global Mean RNFLT** negative correlations with the brain MD when controlling for the CVR factors i.e., BMI, LDL and HDL Cholesterol scores and Diabetes, Hypertension, Smoking and Physical Activity status (**i.e., with CVR**) in addition to age, sex and related retina scan radius **(n=550).** Results shown, at cluster-level corrected p< 0.05 for FWER with an uncorrected p<0.001 voxel-level clustering threshold, on MNI-registered FSL_HCP1065_FA_1mm standard atlas, Neurological View. RNFLT: Retinal Nerve Fiber Layer Thickness, MD: Mean Diffusivity

Right Global RNFLT x Brain MD (-28, 16, 20) without CVR control

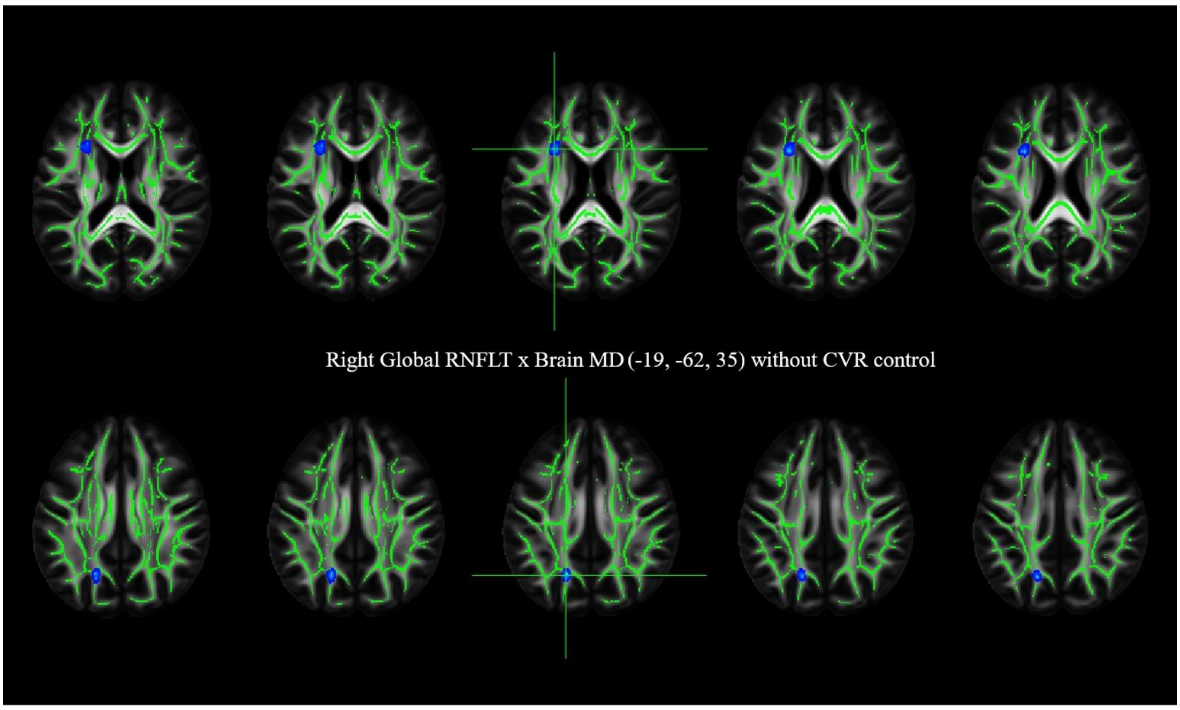

Left Global RNFLT x Brain MD (-28, 16, 20) without CVR control

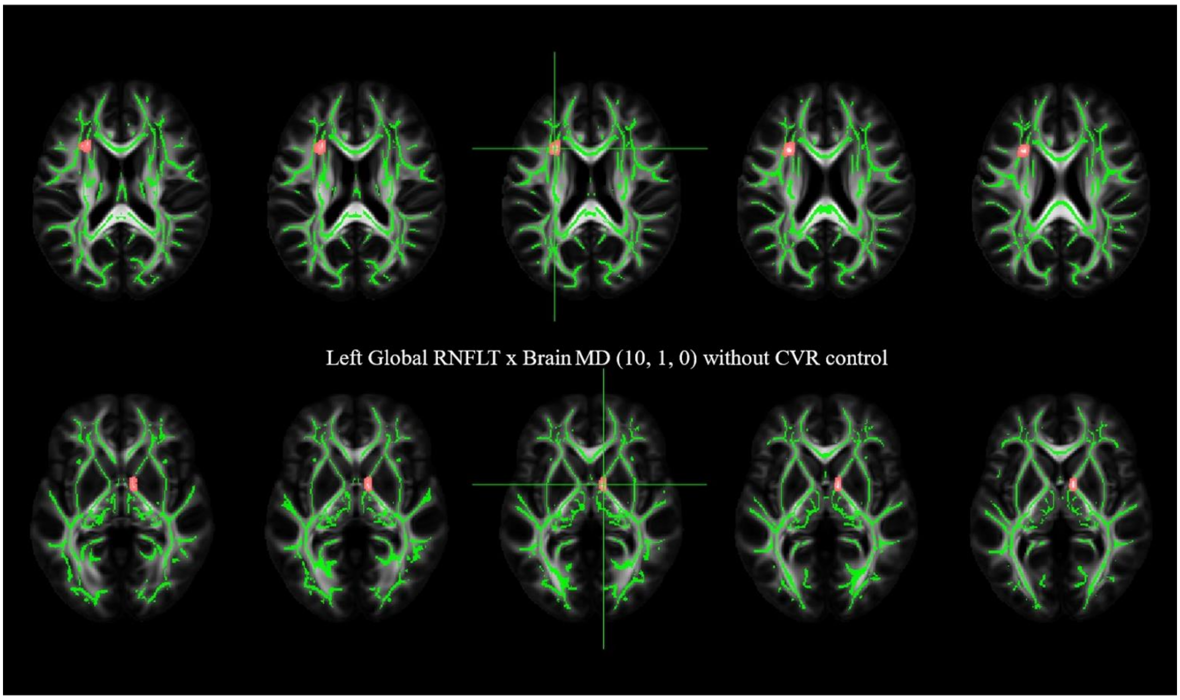

Right and Left Global RNFLT x Brain MD (-28, 16, 20) without CVR control (together)

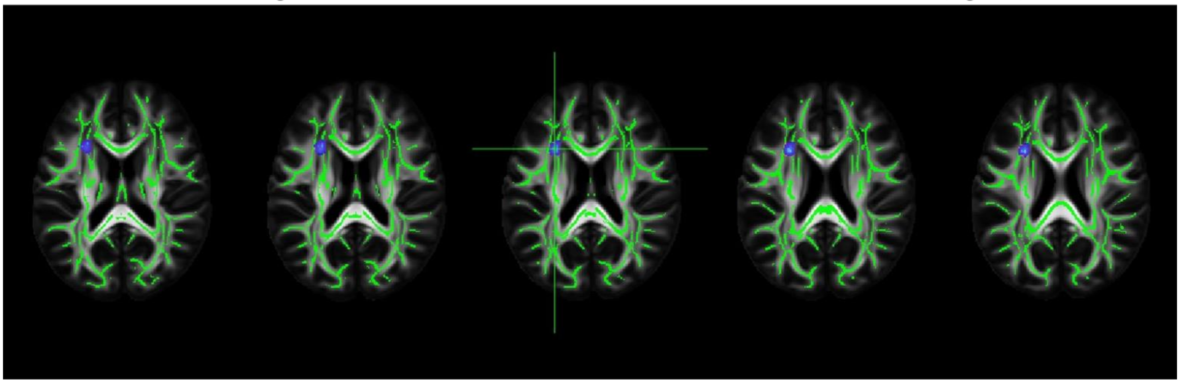

Figure S17. Comparison of the **Right** and **Left Global Mean RNFLT** negative correlations with the brain MD when controlling for only age, sex and regarding retina scan radius [(**i.e., without CVR, (n=550)].** Results shown, at cluster-level corrected p< 0.05 for FWER with an uncorrected p<0.001 voxel-level clustering threshold, on MNI-registered FSL_HCP1065_FA_1mm standard atlas, Neurological View. RNFLT: Retinal Nerve Fiber Layer Thickness, MD: Mean Diffusivity

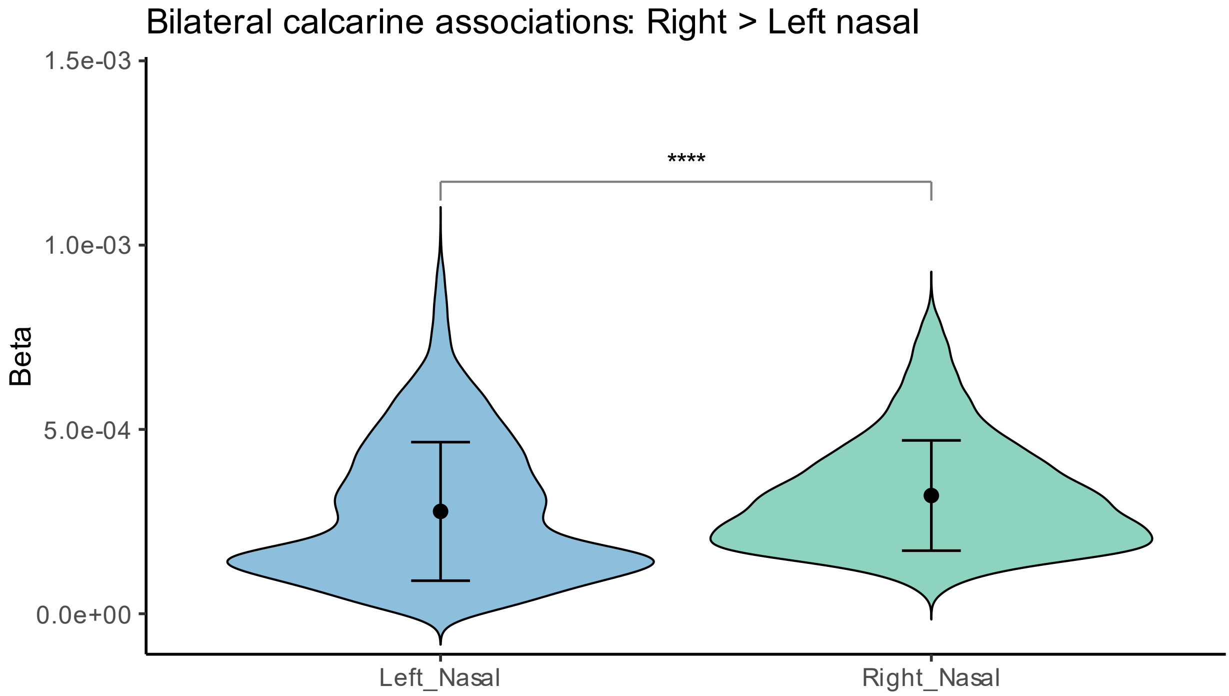

Figure S18. Paired sample t-test results between the Left and Right Nasal RNFLT correlations with the GMD for the Bilateral Calcarine Cortex. RNFLT: Retinal Nerve Fiber Layer Thickness, GMD: gray matter density.

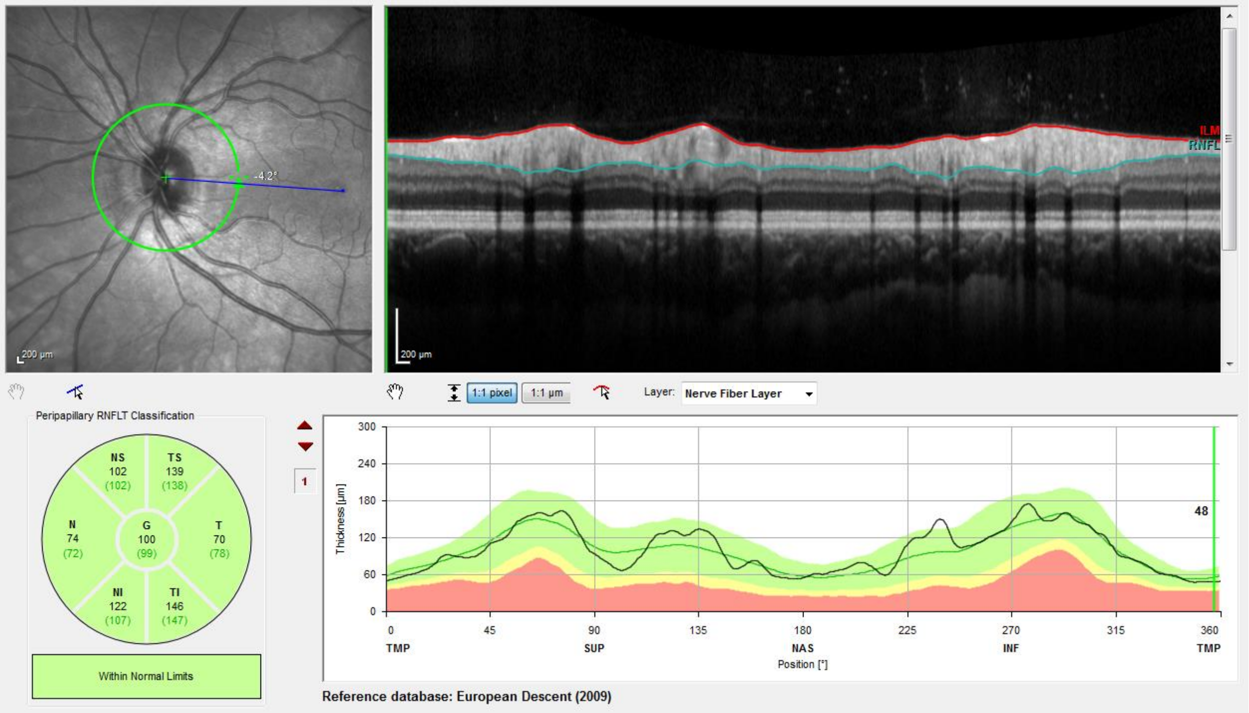

Figure S19. An example image of a circumpapillary RNFLT from an OCT scan (by Franziska G. Rauscher). RNFLT: retinal nerve fibre layer thickness, OCT: optical coherence tomography.

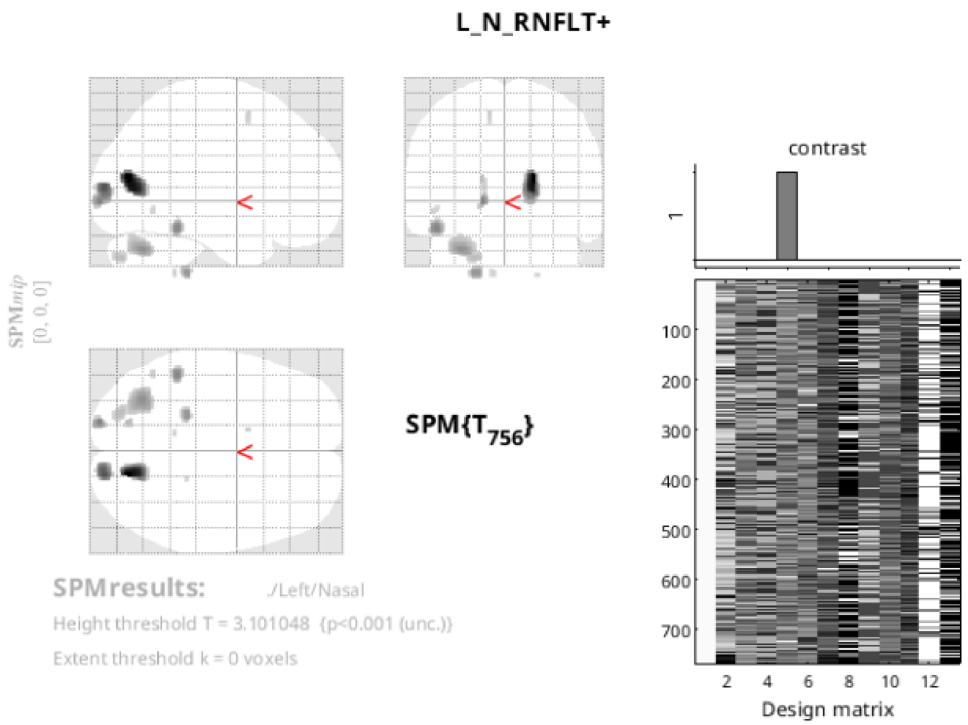

Statistics: ***p-values adjusted for search volume***

set-level		cluster-level				peak-level					mm mm mm		
p	c	$p_{FWE\text{-}corr}$	$q_{FDR\text{-}corr}$	k_E	p_{uncorr}	$p_{FWE\text{-}corr}$	$q_{FDR\text{-}corr}$	T	(Z_E)	p_{uncorr}			
0.021	12	0.378	0.472	333	0.079	0.022	0.047	4.76	4.72	0.000	18	-75	14
		0.552	0.533	235	0.133	0.270	0.339	4.07	4.05	0.000	18	-92	8
		0.949	0.844	49	0.492	0.593	0.534	3.77	3.75	0.000	-16	-94	-2
		0.806	0.816	122	0.272	0.631	0.534	3.73	3.72	0.000	-48	-42	-18
		0.187	0.413	508	0.034	0.753	0.534	3.62	3.61	0.000	-32	-66	-33
		0.923	0.844	65	0.425	0.774	0.534	3.60	3.59	0.000	-21	-36	-48
		0.975	0.919	28	0.614	0.958	0.879	3.34	3.33	0.000	-16	-76	14
		0.897	0.844	79	0.377	0.970	0.879	3.31	3.30	0.000	-22	-81	-39
		0.992	0.919	8	0.809	0.988	0.879	3.22	3.21	0.001	-12	6	56
		0.994	0.919	5	0.857	0.990	0.879	3.21	3.20	0.001	-9	-45	-48
		0.992	0.919	8	0.809	0.994	0.879	3.16	3.15	0.001	-48	-58	-8
		0.996	0.919	2	0.919	0.995	0.879	3.15	3.14	0.001	21	-36	-50

table shows 3 local maxima more than 8.0mm apart

Height threshold: T = 3.10, p = 0.001 (0.998)
Extent threshold: k = 0 voxels
Expected voxels per cluster, <k> = 109.162
Expected number of clusters, <c> = 6.03
FWEp: 4.553, FDRp: 4.759, FWEc: Inf, FDRc: Inf

Degrees of freedom = [1.0, 756.0]
FWHM = 14.8 14.6 14.5 mm mm mm; 9.9 9.8 9.6 {voxels}
Volume: 1489158 = 441232 voxels = 419.0 resels
Voxel size: 1.5 1.5 1.5 mm mm mm; (resel = 929.93 voxels)

Figure S20. Left nasal RNFLT result map at uncorrected p < 0.001 level when all CVRF controlled for in addition to age, sex, TIV and left retina scanning radius in the VBM multiple regression model.

R_N_RNFLT+

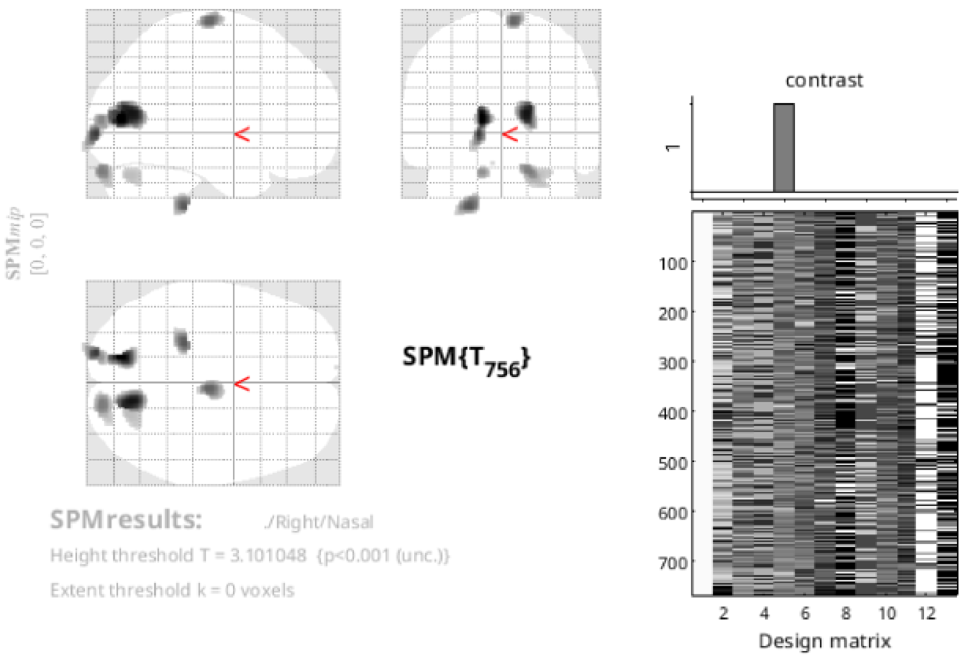

Statistics: ***p-values adjusted for search volume***

set-level		cluster-level				peak-level					mm mm mm		
p	c	$p_{FWE-corr}$	$q_{FDR-corr}$	k_E	p_{uncorr}	$p_{FWE-corr}$	$q_{FDR-corr}$	T	(Z_E)	p_{uncorr}			
0.087	10	0.363	0.264	343	0.075	0.010	0.020	4.94	4.90	0.000	-15	-78	8
		0.141	0.252	579	0.025	0.028	0.028	4.69	4.66	0.000	15	-72	10
		0.688	0.276	173	0.193	0.098	0.051	4.38	4.35	0.000	-18	-96	-3
		0.510	0.264	256	0.118	0.219	0.098	4.14	4.12	0.000	-24	-36	-50
		0.616	0.264	205	0.159	0.257	0.098	4.09	4.07	0.000	6	-20	72
		0.482	0.264	271	0.109	0.466	0.178	3.87	3.85	0.000	16	-92	-26
						0.978	0.686	3.28	3.27	0.001	26	-87	-34
		0.756	0.292	144	0.233	0.574	0.212	3.78	3.76	0.000	18	-92	8
		0.930	0.489	61	0.440	0.627	0.218	3.74	3.72	0.000	-15	-93	-27
		0.571	0.264	226	0.140	0.947	0.584	3.37	3.36	0.000	21	-72	-30
		0.997	0.948	1	0.948	0.997	0.967	3.11	3.10	0.001	-8	68	-21

table shows 3 local maxima more than 8.0mm apart

Height threshold: T = 3.10, p = 0.001 (0.998)
Extent threshold: k = 0 voxels
Expected voxels per cluster, <k> = 109.023
Expected number of clusters, <c> = 6.04
FWEp: 4.553, FDRp: 4.597, FWEc: Inf, FDRc: Inf

Degrees of freedom = [1.0, 756.0]
FWHM = 14.8 14.6 14.5 mm mm mm; 9.9 9.8 9.6 {voxels}
Volume: 1489158 = 441232 voxels = 419.6 resels
Voxel size: 1.5 1.5 1.5 mm mm mm; (resel = 928.74 voxels)

Figure S21. Right nasal RNFLT result map at uncorrected p < 0.001 level when all CVRF controlled for in addition to age, sex, TIV and right retina scanning radius in the VBM multiple regression model.

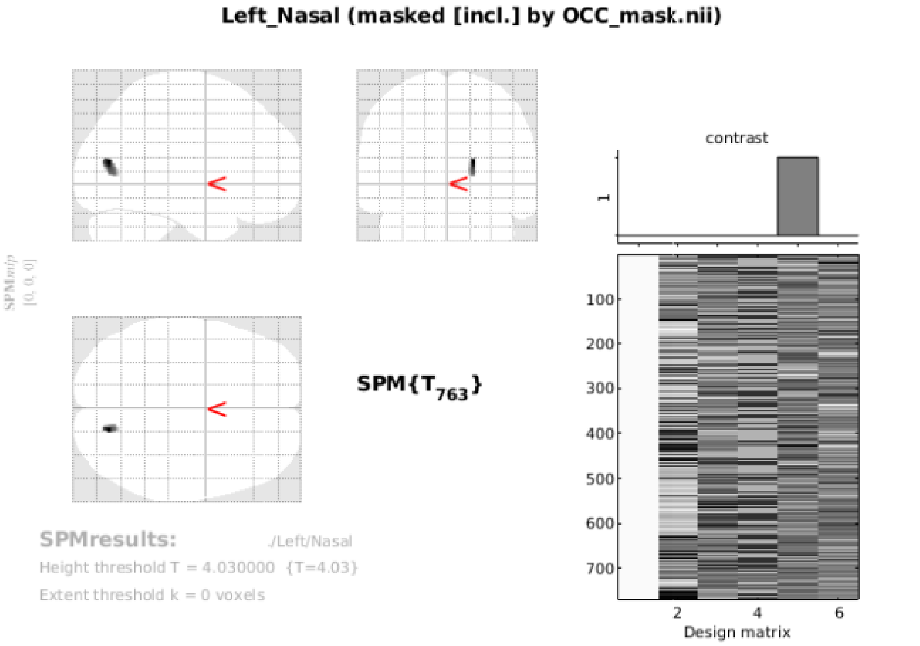

Statistics: ***search volume: image mask: ./OCC_mask.nii***

cluster-level				peak-level					mm mm mm
$p_{FWE-corr}$	$q_{FDR-corr}$	k_E	p_{uncorr}	$p_{FWE-corr}$	$q_{FDR-corr}$	T	(Z_E)	p_{uncorr}	
0.015	0.286	52	0.286	0.007	0.141	4.55	4.51	0.000	18 -75 14

table shows 16 local maxima more than 4.0mm apart

Height threshold: T = 4.03, p = 0.000 (0.050)
Extent threshold: k = 0 voxels
Expected voxels per cluster, <k> = 49.424
Expected number of clusters, <c> = 0.05
FWEp: 4.029, FDRp: Inf, FWEc: 52, FDRc: Inf

Degrees of freedom = [1.0, 763.0]
FWHM = 14.9 14.7 14.5 mm mm mm; 9.9 9.8 9.7 {voxels}
Volume: 156158 = 46269 voxels = 48.4 resels
Voxel size: 1.5 1.5 1.5 mm mm mm; (resel = 938.25 voxels)

Figure S22. Left nasal RNFLT result map with small volume correction at cluster-level pFWE < 0.05 and at uncorrected voxel-level p < 0.001 when all CVRF controlled for in addition to age, sex, TIV and left retina scanning radius in the VBM multiple regression model.

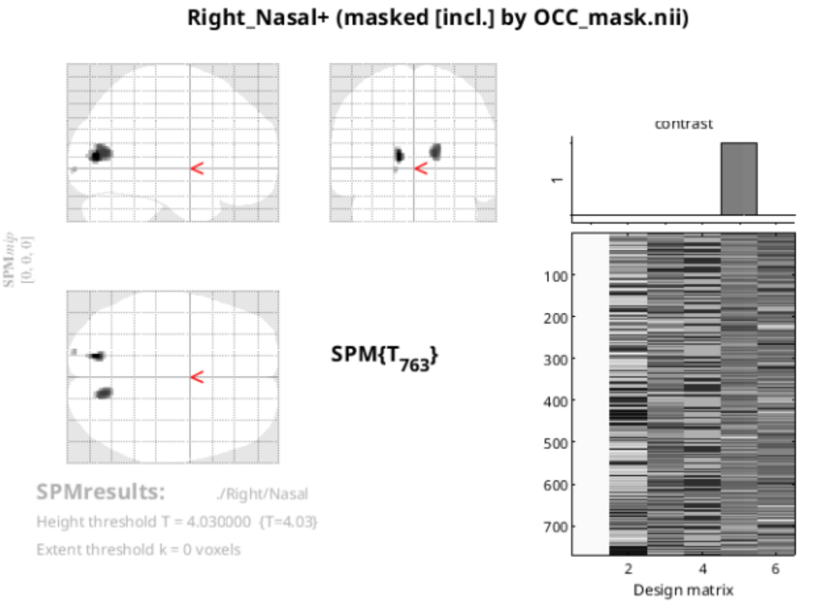

Statistics: *search volume: image mask: ./OCC_mask.nii*

set-level		cluster-level				peak-level					mm mm mm		
p	c	$p_{FWE-corr}$	$q_{FDR-corr}$	k_E	p_{uncorr}	$p_{FWE-corr}$	$q_{FDR-corr}$	T	(Z_E)	p_{uncorr}			
0.000	3	0.007	0.198	107	0.132	0.002	0.154	4.85	4.81	0.000	-15	-78	8
		0.003	0.154	190	0.051	0.005	0.200	4.61	4.58	0.000	15	-72	10
						0.008	0.200	4.53	4.50	0.000	16	-75	14
		0.037	0.743	6	0.743	0.027	0.534	4.20	4.18	0.000	-18	-96	-3

table shows 16 local maxima more than 4.0mm apart

Height threshold: T = 4.03, p = 0.000 (0.050)
Extent threshold: k = 0 voxels
Expected voxels per cluster, <k> = 49.364
Expected number of clusters, <c> = 0.05
FWEp: 4.030, FDRp: Inf, FWEc: 6, FDRc: Inf

Degrees of freedom = [1.0, 763.0]
FWHM = 14.9 14.7 14.5 mm mm mm; 9.9 9.8 9.7 {voxels}
Volume: 156158 = 46269 voxels = 48.4 resels
Voxel size: 1.5 1.5 1.5 mm mm mm; (resel = 937.11 voxels)

Figure S23. Right nasal RNFLT result map with small volume correction at cluster-level pFWE < 0.05 and at uncorrected voxel-level p < 0.001 when only age, sex, TIV and right retina scanning radius were controlled for in the VBM multiple regression model.

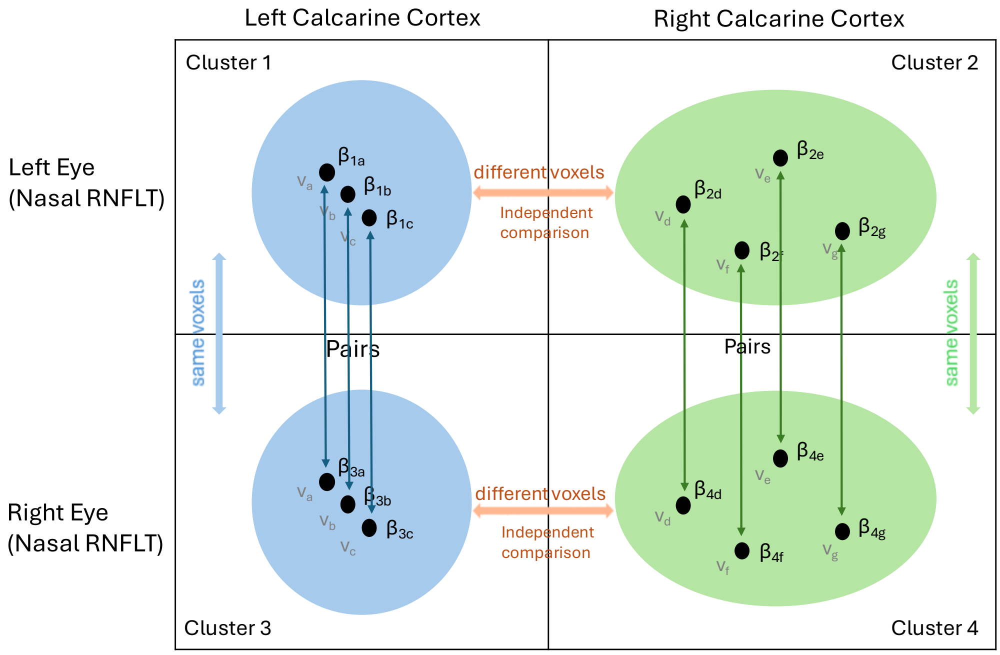

Figure S24. Schematic display of comparison of the voxel-wise population-level beta coefficients (association strengths) used for paired- and independent- samples t-tests

Notes/ Explanations for Figure S24: Rationale for paired and independent comparisons of population-level association strengths

To clarify the statistical rationale underlying the laterality analyses, we provide here an explanation of how paired and independent comparisons were defined in the context of population-level association strengths. These analyses were based on voxel-wise beta coefficients obtained from group-level multiple regression models, which quantify the strength of association between retinal nerve fiber layer thickness (RNFLT) and brain structure across the full sample.

A key point is that pairing in these analyses is defined by voxel identity, rather than by participant identity. Each beta coefficient represents a population-level regression slope estimated for a specific voxel within a given anatomical region. Whether a paired or independent comparison is appropriate therefore depends on whether the same voxels contribute beta coefficients under two different conditions.

The schematic figure illustrates four clusters of beta coefficients, each corresponding to a specific combination of eye (condition) and cortical space. Cluster 1 represents beta coefficients derived from associations between left nasal RNFLT and grey matter density (GMD) within the left calcarine cortex. Cluster 2 represents beta coefficients derived from associations between left nasal RNFLT and GMD within the right calcarine cortex. Cluster 3 represents beta coefficients derived from associations between right nasal RNFLT and GMD within the left calcarine cortex. Cluster 4 represents beta coefficients derived from associations between right nasal RNFLT and GMD within the right calcarine cortex.

Each cluster contains a distribution of voxel-wise beta coefficients extracted from the corresponding anatomical region. Importantly, clusters that belong to the same calcarine cortex (Clusters 1 and 3 for the left calcarine cortex; Clusters 2 and 4 for the right calcarine cortex) are derived from the same set of voxels. As a result, when association strengths are compared between Clusters 1 and 3 or between Clusters 2 and 4, each voxel contributes two beta coefficients corresponding to two eye-specific conditions. These comparisons therefore evaluate within-voxel differences in population-level association strength and are appropriately assessed using paired-sample statistical tests.

In contrast, clusters that belong to different calcarine cortices (Clusters 1 and 2, or Clusters 3 and 4) are derived from anatomically distinct and non-overlapping voxel populations. In these cases, each voxel contributes a single beta coefficient, and there is no one-to-one correspondence between voxels across hemispheres. Consequently, comparisons between these clusters contrast distributions of beta coefficients drawn from different voxel populations and are appropriately assessed using independent-sample statistical tests.

Although left and right calcarine cortex are anatomically linked within the same individuals, the beta coefficients used here are population-level regression parameters estimated separately for each voxel and anatomical region. Therefore, the choice between paired and independent comparisons is determined by voxel identity rather than by participant identity.

Conceptually, these analyses address whether the strength of association between RNFLT and brain structure differs across eyes or across cortical regions. Analogically, like in the case of Fisher's z-transformation of correlation coefficients to be compared across conditions or groups. The schematic figure visually summarizes this logic by illustrating how voxel identity is preserved across eye conditions within the same cortical space, enabling paired comparisons, and how voxel identity differs across cortical spaces, necessitating independent comparisons.

***
