# Supplementary material for: Retinal nerve fibre layer thickness reflects characteristics of brain grey and white matter
Source: Imaging Neurosci (Camb). 2026 Apr 2;4:IMAG.a.1174. doi: 10.1162/IMAG.a.1174 (PMC13058853; doi:10.1162/IMAG.a.1174)
Supplement: Supplementary Tables [file IMAG.a.1174_supp2.pdf]

SUPPLEMENT TABLES

CORRELATIONS

Table S1. Partial Correlations between Average Global RNFLT and CVR factors when controlling for the Average Retina Scan Radius [n=769 (VBM Sample)]

	Age	Sex	Diabetes Status	BMI	Hypertension Status	IPAQ Category	LDL	HDL	Smoking Status
Average Global RNFLT	**-0,27****	-0,11	**-0,12****	-0,06	**-0,1***	-0,04	-0,06	0,05	0,02
Age		0,07	**0,41****	**0,31****	**0,4****	**0,09***	**0,31****	0,03	**-0,12****
Sex			**0,14****	**0,09***	**0,08***	0,04	**0,07***	**-0,46****	**0,13****
Diabetes Status				**0,37****	**0,34****	0,02	0,02	**-0,13****	-0,02
BMI					**0,38****	-0,02	**0,14****	**-0,35****	0,07
Hypertension Status						0,06	0,04	**-0,16****	**-0,09***
IPAQ Category							0,01	0,04	**-0,1***
LDL								**-0,11***	0,03
HDL									**-0,11***

**: p<=0.001, *: p<0.05

Table S2. Partial Correlations between Average Global RNFLT and CVR factors when controlling for the Average Retina Scan Radius [n=550 (TBSS Sample)]

	Age	Sex	Diabetes Status	Hypertension Status	BMI	Smoking Status	IPAQ Category	HDL	LDL
Average Global RNFLT	**-0,28****	-0,07	**-0,15****	**-0,11***	-0,02	**0,08***	-0,04	0,003	**-0,11***
Age		0,04	**0,39****	**0,39****	**0,31****	**-0,16****	**0,1***	0,04	**0,37****
Sex			**0,14****	0,02	0,06	**0,13***	0,04	**-0,47****	**0,1***
Diabetes Status				**0,32****	**0,33****	-0,02	0,04	**-0,13***	**0,09***
Hypertension Status					**0,34****	**-0,09***	0,07	**-0,11***	0,08
BMI						**0,09***	-0,04	**-0,32****	**0,2****
Smoking Status							-0,08	**-0,14****	0,02
IPAQ Category								0,04	0,01
HDL									**-0,15****

**: p<=0.001, *: p<0.05

RESULTS: VBM

Table S3. Whole Brain VBM Results: Average, Left and Right Global Mean RNFLT positive correlations with the brain gray matter density (when only age, sex, TIV and retina scan radius controlled)

RNFLT					coordinates mm			
	cluster p(FWE-corr)	cluster size	peak T	peak p(unc)	x	y	z	Area*
Average Global	0,008	1421	6,07	0,000	16	-80	14	R Calcarine Cortex/ V1, 17 CalcS, hOc1
			5,3	0,000	18	-90	4	
	0,009	1358	6	0,000	-15	-78	8	L Calcarine Cortex/ V1, 17 CalcS, hOc1
			5,15	0,000	-16	-94	0	
Left Global	0,019	1137	5,67	0,000	16	-78	14	R Calcarine Cortex/ V1, 17 CalcS, hOc1
			5,05	0,000	18	-90	4	
	0,038	936	5,53	0,000	-15	-78	8	L Calcarine Cortex/ V1, 17 CalcS, hOc1
			4,63	0,000	-16	-94	0	
Right Global	0,006	1500	6,02	0,000	16	-80	14	R Calcarine Cortex/ V1, 17 CalcS, hOc1
			5,75	0,000	16	-70	14	
			5,2	0,000	20	-90	3	
	0,004	1666	5,99	0,000	-15	-80	8	L Calcarine Cortex/ V1, 17 CalcS, hOc1
			5,32	0,000	-16	-94	0	
			3,75	0,000	-6	-88	4	

*: CAT12 Neuromorphometrics Atlas

					coordinates mm		
	voxel p(FWE-corr)	cluster size	peak T	peak p(unc)	x	y	z
Left Nasal	0,022	15	4,76	0	18	-75	14
Right Nasal	0,01	32	4,94	0	-15	-78	8
	0,028	34	4,69	0	15	-72	10

T-test Findings

Table S4. Paired-t test results between the Right Global RNFLT correlations (beta coefficients) with the GMD and the Left Global RNFLT correlations (beta coefficients) with the GMD in Bilateral Calcarine Cortex

Mean difference (Left-Right)	T-statistic*	p-value	Degrees of Freedom	%95 CI-low	%95 CI-high	Cohen's_d
-0.000043	-38.8	3.64E-303	8098	-Inf	-0.0000412	-0.43

* One sided paired-t-test, Alternative H: The Right RNFLT correlations are greater than the Left RNFLT correlations, RNFLT correlations from the whole-brain VBM when all CVR factors were controlled for the bilateral Calcarine ROI taken from the Neuromorphometrics Atlas.

Table S5. Paired-t test results between the Left Nasal RNFLT correlations (+ beta coefficients) and the Right Nasal RNFLT correlations (+ beta coefficients) with the GMD in the **Left Calcarine Cortex**

Mean difference (Left_N_leftCalc-Right_N_leftCalc)	T-statistic*	p-value	Degrees of Freedom	%95 CI-low	%95 CI-high	Cohen's_d
-0.0000994491	-111.97	0	4480	-Inf	-0.0000979879	-1.67271

* One sided paired-t-test, Alternative H: The Left Nasal RNFLT correlations are less than the Right Nasal RNFLT correlations with the Left Calcarine GMD, RNFLT correlations from the whole-brain VBM when all CVR factors were controlled.

Table S6. Paired-t test results between the Left Nasal RNFLT correlations (+ beta coefficients) and the Right Nasal RNFLT correlations (+ beta coefficients) with the GMD in the **Right Calcarine Cortex**

Mean difference (Left_N_rightCalc-Right_N_rightCalc)	T-statistic*	p-value	Degrees of Freedom	%95 CI-low	%95 CI-high	Cohen's_d
0.0000268147	16.943	2.51042997772905E-062	3617	0.0000242108	Inf	0.281685

* One sided paired-t-test, Alternative H: The Left Nasal RNFLT correlations are greater than the Right Nasal RNFLT correlations with the Right Calcarine GMD, RNFLT correlations from the whole-brain VBM when all CVR factors were controlled.

Table S7. Independent-sample t-test results between the **Left Nasal** RNFLT correlations (+ beta coefficients) with the GMD in the left and the Right Calcarine Cortex

Mean Left_N_leftCalc	Mean Left_N_rightCalc	T-statistic*	Degrees of Freedom	p-value	%95 CI-low	%95 CI-high	Cohen's_d
0.000162485	0.000420177	-80.743	6220.549285	0	-Inf	-0.000252441	-1.87315

* One sided independent-t-test (Welch's Two Sample T-test), Alternative H: The Left Nasal RNFLT correlations with the left Calcarine GMD are less than the Left Nasal RNFLT correlations with the right Calcarine GMD, RNFLT correlations from the whole-brain VBM when all CVR factors were controlled.

Table S8. Independent-sample t-test results between the **Right Nasal** RNFLT correlations (+ beta coefficients) with the GMD in the left and the Right Calcarine Cortex

Mean Right_N_leftCalc	Mean Right_N_rightCalc	T-statistic*	Degrees of Freedom	p-value	%95 CI-low	%95 CI-high	Cohen's_d
0.000248586	0.000393362	-48.683	7639.357902	1	-0.000149669	Inf	-1.07148

* One sided independent-t-test (Welch's Two Sample T-test), Alternative H: The Right Nasal RNFLT correlations with the left Calcarine GMD are greater than the Right Nasal RNFLT correlations with the right Calcarine GMD, RNFLT correlations from the whole-brain VBM when all CVR factors were controlled.

Table S9. Paired-t test results between the Left Nasal RNFLT correlations (beta coefficients) and the Right Nasal RNFLT correlations (beta coefficients) with the GMD in the **Bilateral Calcarine Cortex**

Mean difference (Left_N_bilCalc-Right_N_bilCalc)	T-statistic *	p-value	Degrees of Freedom	%95 CI-low	%95 CI-high	Cohen's_d
-4.883567e-05	-44.873	< 2.2e-16	8545	-Inf	-0.0000470	-0.2544713

* One sided paired-t-test, Alternative H: The Left Nasal RNFLT correlations are less than the Right Nasal RNFLT correlations with the Bilateral Calcarine Cortex GMD, RNFLT correlations from the whole-brain VBM when all CVR factors were controlled.

CVRF: VBM Findings

Table S10. Whole Brain VBM Results: **BMI, Diabetes and Smoking** negative correlations with the brain gray matter density (when age, sex and TIV controlled for)

						coordinates (mm)	
	cluster p(FWE-corr)	cluster size	peak T	peak p(unc)	x	y	z
BMI	0	6794	6,29	0	6	-15	-2
			5,73	0	-6	-10	0
			3,99	0	21	-32	-14
	0,007	1467	5,51	0	39	-20	54
			3,91	0	30	-30	56
	0	7070	5,47	0	-27	-72	-44
			5,43	0	-39	-58	-63
			5,32	0	-30	-74	-60
	0,016	1183	5,19	0	28	52	27
			3,35	0	10	58	28
	0	5000	5,16	0	27	68	-15
			4,65	0	32	66	3
			4,65	0	6	64	-20
	0	3057	5,16	0	34	-64	-62
			4,91	0	22	-75	-58
			4,56	0	12	-80	-52
	0,039	926	5,11	0	-32	42	38
			4,54	0	-33	30	40
			4,15	0	-30	30	51
	0	2598	4,69	0	-40	16	-3
			4,66	0	-46	10	10
			4,48	0	-27	9	-14
Diabetes	0,001	2201	5,07	0	48	-12	54
			4,24	0	50	-22	51
			3,83	0	54	-10	32
	0,005	1577	4,64	0	21	-50	-12
			4,08	0	14	-68	-6
			4,05	0	16	-60	-9
	0,003	1723	4,63	0	63	-21	-24
			4,08	0	69	-18	-6

	0,043	900	4,58	0	-20	-45	-8
			3,28	0,001	-14	-38	2
	0,034	966	3,94	0	-28	-27	74
			3,82	0	-20	-27	68
			3,82	0	-38	-34	60
Smoking	0,023	1086	4,57	0	-21	-66	-8
			4,22	0	-28	-60	-14
			4,05	0	-22	-72	-15

Table S11. Whole Brain VBM Results: **HDL and LDL cholesterol** positive correlations with the brain gray matter density (when age, sex and TIV controlled for)

						coordinates (mm)	
	cluster p(FWE-corr)	cluster size	peak T	peak p(unc)	x	y	z
HDL	0,005	1539	4,55	0	-46	-20	-38
			4,3	0	-58	-15	-40
			4,07	0	-40	-9	-48
	0,001	2336	4,41	0	34	-62	-54
			4,12	0	27	-74	-58
			3,77	0	33	-69	-46
	0,003	1690	3,87	0	-21	-76	-46
			3,86	0	-18	-86	-51
			3,38	0	-27	-84	-34
LDL	0,002	1898	5,67	0	32	-87	20
			4,17	0	28	-88	0
			3,71	0	28	-98	15
	0,044	893	4,46	0	38	38	-9
			3,48	0	20	39	-16
	0	2951	4,43	0	-39	-21	3
			4,18	0	-20	-60	0
			4	0	-22	-22	-10
	0,002	1935	4,42	0	0	-51	28
			3,91	0	10	-32	34
			3,44	0	2	-24	28
	0,015	1204	4,32	0	-68	-26	-6
			4	0	-64	-34	0
			3,37	0	-57	-46	-2

RESULTS: TBSS

Table S12. Whole Brain TBSS Results: Left and Right Global Mean RNFLT positive correlations with the brain **Fractional Anisotropy** (**with** CVRF controlling)

			coordinates			
	Cluster Size	pFWE -corr	X (mm)	Y (mm)	Z (mm)	
Left	108	0,005	-29	-63	-1	IFOF+ILF+*Fmaj*/ PTR[OR]/ OR+CC+Visual Cortex
	80	0,012	-30	-11	-14	ILF+*ATR+IFOF*/ unc+*Fornix+Stria Terminalis*/ *OR*+Amygdala+Hippocampus+OR
	78	0,012	34	-14	-13	ILF/ Fornix+Stria Terminalis/ *OR*+Hippocampus+OR+Fornix+*LGN+AcRad*
	49	0,039	18	-80	-1	IFOF+ILF+FMaj/ unc/ OR+Visual Cortex
Right	140	0,002	-38	-44	-8	ILF+IFOF/ Sagittal Stratum[ILF+IFOF]+*PTR[OR]*/ OR+CC
	107	0,004	-31	-64	-1	IFOF+ILF/ PTR[OR]+*Sagittal Stratum[ILF+IFOF]/* OR+CC+Visual Cortex
	106	0,004	40	-36	-11	ILF+IFOF/ Sagittal Stratum[ILF+IFOF]/ OR+CC
	100	0,004	-32	-13	-14	-+*ATR*/Fornix+Stria Terminalis/ *OR*+Hippocampus+OR+Amygdala
	90	0,006	34	-10	-15	-/Fornix+Stria Terminalis/ Hippocampus+Amygdala+OR+Fornix+*AcRad*
	82	0,009	31	-51	13	Fmaj+IFOF/ Tapetum/ CC
	74	0,011	25	-37	-8	Cingulum (hippocampus)/ unc+*Cingulum (hippocampus)*/ Hippocampus+OR+CC+*Cingulum*

Table S13. Whole Brain TBSS Results: Average, Left and Right Global Mean RNFLT positive correlations with the brain **Fractional Anisotropy** (**without** CVRF controlling)

			coordinates			
RNFLT	Cluster size	pFWE -corr	x	y	z	Tract/ Area
avG_woCVR	104	0,006	-31	-64	-1	IFOF+ILF+*Fmaj*/ PTR[OR]/ OR+CC+Visual Cortex
	90	0,009	34	-10	-15	-/ Fornix+Stria Terminalis/ *OR*+**Hippocampus**+Amydala+OR+Fornix+*LGN+AcRad*
	86	0,01	-34	-15	-13	IFOF+*ATR*/ unc+*Fornix+Stria Terminalis*/ *OR*+OR+IFOF
	79	0,012	-38	-44	-8	IFOF+ILF/ Sagittal Stratum[ILF+IFOF]/ OR+CC
	65	0,02	18	-80	-1	IFOF+ILF+Fmaj/ unc/ Visual Cortex+OR

	61	0,022	34	-59	0	IFOF+ILF/ PTR[OR]/ CC+OR+Visual Cortex
	49	0,037	31	-52	13	Fmaj+IFOF/ Tapetum/ CC+Visual Cortex
Left_G_woCVR	86	0,01	-24	-75	0	IFOF+ILF+Fmaj/ unc+*PTR[OR]*/ OR+Visual Cortex+CC
	71	0,015	34	-14	-13	IFOF/ Fornix+Stria Terminalis/ Hippocampus+OR+Fornix+*LGN+AcRad*
	65	0,019	-30	-11	-14	ILF+*ATR*/ unc+*Fornix+Stria Terminalis*/ Amydala+Hippocampus+OR
	50	0,039	18	-80	-1	IFOF+ILF+Fmaj/ unc/ OR+Visual Cortex+CC
Right_G_woCVR	117	0,004	-38	-44	-8	IFOF+ILF/ Sagittal Stratum[ILF+IFOF]+*PTR[OR]*/ OR+CC
	103	0,005	-31	-64	-1	IFOF+ILF+*FMaj*/ PTR[OR]/ OR+CC+Visual Cortex
	89	0,008	-31	-14	-13	-+*ATR*/ Fornix+Stria Terminalis/ OR+Hippocampus+Amydala
	87	0,009	34	-10	-15	-/ Fornix+Stria Terminalis/ **Hippocampus**+Amydala+OR+Fornix+*Ac Rad*
	80	0,011	31	-51	13	Fmaj+IFOF/ Tapetum/ CC
	78	0,011	25	-37	-8	Cingulum (hippocampus)/ unc+*Cingulum (hippocampus)/* Hippocamous+OR+CC+*Cingulum*
	72	0,014	40	-36	-11	ILF+IFOF/ Sagittal Stratum[ILF+IFOF]/ OR+CC+*Fornix*

T-Test Finding

Table S14. Paired-t test results between the Right Global RNFLT correlations with the **Fractional Anisotropy** and the Left Global RNFLT correlations with the **Fractional Anisotropy** in Optic Radiata

Mean difference (Right-Left)	T-statistic*	p-value	Degrees of Freedom	%95 CI-low	%95 CI-high	Cohen's_d
0.0008869154	39.16	2.2e-16	199583	0.0008496655	inf	0.09

* One sided paired-t-test, Alternative H: The Right global RNFLT correlations are greater than the Left global RNFLT correlations, RNFLT correlations from the whole-brain TBSS when all CVR factors were controlled for the bilateral Optic Radiation ROI obtained from Juelich Histological Atlas multiplied with the mean FA skeleton.

TBSS: Mean Diffusivity

Table S15. Whole Brain TBSS Results: Average Global Mean RNFLT negative correlations with the brain **Mean Diffusivity** (**without** CVRF controlling)

			coordinates			
	Cluster size	pFWE-corr	x	y	z	Tract/Area
avG_MD_woCVR	44	0,021	-30	18	18	-/unc/Broca's Area/IFG(Harvard)/Insula(MNI)

Table S16. Whole Brain TBSS Results: **Left** and **Right** Global Mean RNFLT negative correlations with the brain **Mean Diffusivity** (**with** CVRF controlling)

	Voxels	pFWE-corr	MAX X (mm)	MAX Y (mm)	MAX Z (mm)	
Left_G_MD	37	0,025	-30	18	18	-/unc/Broca's Area/IFG(Harvard)/Insula(MNI)
Right_G_MD	39	0,023	-30	18	18	-/unc/Broca's Area/IFG(Harvard)/Insula(MNI)

CVRF: TBSS

Table S17. Whole Brain TBSS Results Hypertension and Smoking negative correlations with the brain Fractional Anisotropy (when controlling for age and sex)

CVR Factor				coordinates		
	Cluster size	pFWE-corr	x	y	z	Tract/Area
Hypertension_FA	42	0,052	-42	-59	18	Inferior Parietal Lobule
Smoking_FA	67	0,018	-28	-47	32	SLF/SLF/IPS+SLF+*OR*+*SSC(I)*

Table S18. Whole Brain TBSS Results Hypertension and Diabetes positive correlations with the brain Mean Diffusivity (when controlling for age and sex)

CVR Factor	Cluster Size	pFWE-corr	x	y	z	Tract/Area
	157	0,001	-15	26	17	Forceps minor+*Cingulum(%1)*/CC/CC
	109	0,003	-13	-24	28	-/SCR/CC+CST
	99	0,004	-10	2	27	-/CC/CC+Cingulum

Hypertension_ MD_pos	87	0,005	30	32	6	IFOF/-/-SLF(%2)
	76	0,007	-20	23	27	SLF(%2)+UF(%1)/ACR/CC
	65	0,011	-18	-2	35	-/SCR/CC+*PremotorC*(%2)
	64	0,011	-34	-8	20	SLF/SLF/SSC(II)[Parietal Operculum]+CST(%2)+SSC(I)+*SSC(II)%1 +PMC%1*
	54	0,015	-20	44	7	Forceps minor+ATR+*IFOF(%4)+Cingulum(%2)+ UF(%1)*/-/CC
	38	0,031	-33	-1	7	SLF/ExtCapsule/-
	36	0,035	-17	-29	29	-/CC/CC
	35	0,037	-19	-52	20	Fmajor/CC/CC+*OR*
	33	0,041	14	11	26	-/CC/CC+*Cingulum(%1)*
	32	0,044	-35	23	15	*IFOF+ILF(%1)+UF(%1)*/-/Broca44+*45*
	31	0,048	19	-13	43	-/-/CST+PremotorC
Diabetes_MD _pos	52	0,016	7	-23	24	-/CC/**CC**+Fornix+*Cingulum*

Table S19. Whole Brain TBSS Results BMI positive and negative correlations with the brain Mean Diffusivity (when controlling for age and sex)

			coordinates			
	Cluster size	pFWE _corr	x	y	z	Tract/Area
MD_pos	40	0,032	-14	33	6	Fminor+*Cingulum*/CC/CC
MD_neg	306	0	6	-20	-30	CST+*ATR*(%2)/CST/-
	73	0,005	41	-60	-10	ILF(%2)/-/VisualC(%2)+OR
	65	0,007	-5	-19	-29	CST/MiddleCerebellar Pedincle/-
	56	0,01	28	-62	24	IFOF+[*SLF%1+ILF%1+FMajor%2*]/-/OR+CC+*SPL*(%1)
	53	0,011	33	-57	29	ILF(%2)+*SLF*(%2)/-/OR+IPS+*OR*(%3)
	52	0,011	38	-19	-9	IFOF+ILF/Sagittal Stratum[ILF+IFOF]/OR+Insula+AR+Hipp(%2)+*IFOF(%1)+CC(%1)*
	43	0,017	-30	-85	-6	IFOF(%2)+*ILF*(%1)/-/VisualCortex+OR
	42	0,019	32	-66	-9	ILF+IFOF+*Cingulum(Hipp%1*)/-/VisualCortex+OR+*CC*(%4)
	38	0,025	36	-31	-21	-*ILF*(%4)+*Cingulum(Hipp%1*)/-/Hippocampus+**OR**
	31	0,036	6	-17	-7	ATR/-/-
	29	0,041	33	25	-14	---*IFOF*(%1)
	29	0,041	-30	-59	-12	ILF+IFOF/-/OR
	29	0,041	33	-57	18	ILF+IFOF+FMajor(%4)+SLF(%1)/PTR[OR]/ OR+CC
	27	0,049	29	-16	-26	Cingulum(Hipp)/Hippocampus/**Hippocampus**

Table S20. Partial correlation coefficients [for pre-chosen **ROIs** below, from the literature, the number of ROIs was 49 in total] between the average Global RNFLT and brain regional gray/white matter volume measures (age, sex and retina scan radius were always controlled for)

**RNFLT correlations**					
**without CVRF correction**			**with CVRF correction**		
	**raw**	**FDR corrected**		**raw**	**FDR corrected**
Whole-brain GM	r=0.044, p=0.226		Whole-brain GM	r=0.049, p=0.179	
Whole-brain WM	r=0.100, p=0.006**	p=0.058	Whole-brain WM	r=0.101, p=0.005**	p=0.048*
Right Caudate	r=0.035, p=0.337		Right Caudate	r=0.035, p=0.297	
Left Caudate	r=0.040, p=0.265		Left Caudate	r=0.044, p=0.228	
Right Cerebellum	r=0.081, p=0.025*	p=0.113	Right Cerebellum	r=0.086, p=0.018*	p=0.096
Left Cerebellum	r=0.102, p=0.005**	p=0.058	Left Cerebellum	r=0.107, p=0.003**	p=0.036*
Right Cerebellar WM	r=-0.033, p=0.358		Right Cerebellar WM	r=-0.032, p=0.384	
Left Cerebellar WM	r=-0.021, p=0.569		Left Cerebellar WM	r=-0.018, p=0.625	
Right Hippocampus	r=0.069, p=0.057		Right Hippocampus	r=0.067, p=0.064	
Left Hippocampus	r=0.046, p=0.207		Left Hippocampus	r=0.044, p=0.220	
Right Pallidum	r=0.065, p=0.074		Right Pallidum	r=0.062, p=0.086	
Left Pallidum	r=0.068, p=0.059		Left Pallidum	r=0.066, p=0.070	
Right Putamen	r=0.045, p=0.214		Right Putamen	r=0.045, p=0.217	
Left Putamen	r=0.057, p=0.113		Left Putamen	r=0.058, p=0.110	
Right Thalamus	r=0.010, p=0.779		Right Thalamus	r=0.015, p=0.671	

Left Thalamus	r=-0.008, p=0.816		Left Thalamus	r=-0.004, p=0.908	
Optic Chiasm	r=-0.050, p=0.165		Optic Chiasm	r=-0.055, p=0.128	
Right Ant. Cingulate	r=0.035, p=0.330		Right Ant. Cingulate	r=0.039, p=0.285	
Left Ant. Cingulate	r=-0.021, p=0.558		Left Ant. Cingulate	r=-0.018, p=0.614	
Right Calcarine	r=0.186, p=0.000***	p=0.000***	Right Calcarine	r=0.190, p=0.000***	p=0.000***
Left Calcarine	r=0.165, p=0.000***	p=0.000***	Left Calcarine	r=0.171, p=0.000***	p=0.000***
Right Cuneus	r=0.041, p=0.263		Right Cuneus	r=0.040, p=0.273	
Left Cuneus	r=0.041, p=0.253		Left Cuneus	r=0.042, p=0.248	
Right Entorhinal	r=0.026, p=0.468		Right Entorhinal	r=0.024, p=0.500	
Left Entorhinal	r=0.057, p=0.116		Left Entorhinal	r=0.056, p=0.121	
Right Fusiform	r=0.031, p=0.390		Right Fusiform	r=0.033, p=0.368	
Left Fusiform	r=0.067, p=0.064	p=0.219	Left Fusiform	r=0.072, p=0.047*	p=0.188
Right Lingual	r=0.081, p=0.026*	p=0.113	Right Lingual	r=0.083, p=0.022*	p=0.096
Left Lingual	r=0.041, p=0.255		Left Lingual	r=0.043, p=0.236	
Right Inf. Occipital	r=-0.029, p=0.429		Right Inf. Occipital	r=-0.028, p=0.445	
Left Inf. Occipital	r=0.022, p=0.535		Left Inf. Occipital	r=0.023, p=0.519	
Right Mid. Cingulate	r=-0.021, p=0.561		Right Mid. Cingulate	r=-0.020, p=0.588	
Left Mid. Cingulate	r=-0.086, p=0.017*	p=0.101	Left Mid. Cingulate	r=-0.083, p=0.022*	p=0.096
Right Mid. Occipital	r=0.024, p=0.499		Right Mid. Occipital	r=0.024, p=0.514	
Left Mid. Occipital	r=-0.006, p=0.862		Left Mid. Occipital	r=-0.006, p=0.876	
Right Mid. Temporal	r=0.033, p=0.363		Right Mid. Temporal	r=0.036, p=0.325	
Left Mid. Temporal	r=0.011, p=0.763		Left Mid. Temporal	r=0.013, p=0.717	
Right Occipital Pole	r=0.039, p=0.279		Right Occipital Pole	r=0.039, p=0.283	

Left Occipital Pole	r=0.085, p=0.019*	p=0.101	Left Occipital Pole	r=0.085, p=0.019*	p=0.096
Right Occ. Fusiform	r=0.043, p=0.237		Right Occ. Fusiform	r=0.044, p=0.224	
Left Occ. Fusiform	r=0.028, p=0.436		Left Occ. Fusiform	r=0.031, p=0.393	
Right Sup. Occipital	r=0.060, p=0.097		Right Sup. Occipital	r=0.060, p=0.098	
Left Sup. Occipital	r=0.034, p=0.354		Left Sup. Occipital	r=0.034, p=0.342	
Right LGN	r=0.027, p=0.464		Right LGN	r=0.029, p=0.423	
Left LGN	r=-0.002, p=0.945		Left LGN	r=0.000, p=0.997	
Right Post. Cingulate	r=-0.005, p=0.892		Right Post. Cingulate	r=-0.007, p=0.870	
Left Post. Cingulate	r=-0.050, p=0.168		Left Post. Cingulate	r=-0.052, p=0.154	
Right Parahippocampal	r=0.086, p=0.017*	p=0.101	Right Parahippocampal	r=0.087, p=0.017*	p=0.096
Left Parahippocampal	r=0.084, p=0.019*	p=0.101	Left Parahippocampal	r=0.087, p=0.016*	p=0.096

***
